# ScHiCAtt: Enhancing single-cell Hi-C data resolution using attention-based models

**DOI:** 10.1016/j.csbj.2025.02.031

**Published:** 2025-02-27

**Authors:** Rohit Menon, H.M.A. Mohit Chowdhury, Oluwatosin Oluwadare

**Affiliations:** a Department of Computer Science, University of Colorado at Colorado Springs, Colorado Springs, 80918, CO, USA; b Department of Biomedical Informatics, University of Colorado Anschutz Medical Campus, Aurora, 80045, CO, USA

**Keywords:** Hi-C data, Self-attention, Resolution enhancement, Single-cell Hi-C, Data sparsity

## Abstract

The spatial organization of chromatin is fundamental to gene regulation and essential for proper cellular function. The Hi-C technique remains one of the leading methods for unraveling 3D genome structures; however, limited resolution, data sparsity, and incomplete coverage in single-cell Hi-C data pose significant challenges for comprehensive analysis. Traditional convolutional neural network-based models often suffer from blurring and loss of fine details, while generative adversarial network based methods encounter difficulties in maintaining diversity and generalization. Moreover, existing algorithms perform poorly in cross-cell line generalization, where a model trained on one cell type is used to enhance high-resolution data in another cell type. To address these limitations, we propose ScHiCAtt (Single-cell Hi-C Attention-Based Model), which leverages attention mechanisms to capture both long-range and local dependencies in Hi-C data, significantly enhancing resolution while preserving biologically meaningful interactions. By dynamically focusing on regions of interest, attention mechanisms effectively mitigate data sparsity and enhance model performance in low-resolution contexts. Extensive experiments on Human and Drosophila single-cell Hi-C data demonstrate that ScHiCAtt consistently outperforms existing methods in terms of computational and biological reproducibility metrics across various downsampling ratios. Our results also show superior generalization across different chromosomes of the same cell type, as well as across cell types, species, and from single-cell to bulk Hi-C data, highlighting the robustness and adaptability of our approach. ScHiCAtt source code is publicly available at https://github.com/OluwadareLab/ScHiCAtt.

## Graphical abstract

**Figure:**
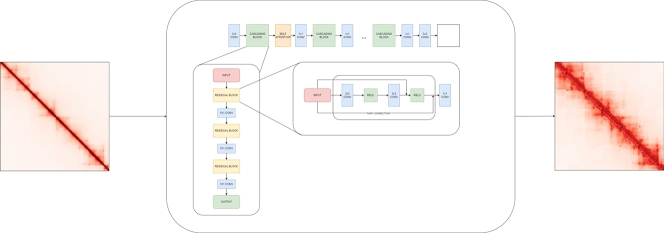

## 1 Introduction

Three-dimensional (3D) conformation of chromosomes is crucial for elucidating genomic processes within the eukaryotic cells' nuclei. The Hi-C technique facilitates an all-versus-all mapping of chromosomal fragment interactions, resulting in an interaction frequency contact matrix, n×n, where *n* represents the number of fragments in a chromosome or genome at a specific resolution, [14]. These Hi-C data are critical for numerous algorithms designed to improve the understanding of genome organization, [18]. A major challenge in this field is the scarcity of high-resolution Hi-C data, which are indispensable for identifying intricate genomic topologies such as enhancer-promoter interactions and subdomains. To address this need, deep learning models have been employed to predict high-resolution data from low-resolution data with remarkable accuracy. Notable models in this area include HiCPlus [29], HiCNN [16], hicGAN [15], Boost-HiC [3], HiCSR [5], SRHiC [13], HiCNN2 [17], HiCARN [8], and DeepHiC [9]. These models leverage various network architectures such as Convolutional Neural Networks (CNNs), Autoencoders, and Generative Adversarial Networks (GANs). Despite the advancements made by these models, there remains considerable room for improvement, especially when it comes to single-cell Hi-C data enhancement, [25], as all of the aforementioned methods are designed for bulk Hi-C data enhancement.

Single-cell Hi-C (scHi-C) is a groundbreaking technology that offers a unique opportunity to investigate 3D genome structures at the single-cell level with high resolution, [7]. By capturing chromatin interactions at this level, scHi-C enables the exploration of cellular heterogeneity in chromatin conformation, [2], [4], [20]. However, scHi-C data are characterized by high dimensionality, noise, and sparsity, presenting computational challenges that demand innovative solutions for the accurate reconstruction of 3D genome structures, [19], [7]. Therefore, scHi-C data imputation is crucial, as it enables the reconstruction of enhanced contact maps from raw and sparse scHi-C data, thereby improving the quality for downstream analyses, including the reconstruction of chromatin organization at the single-cell level. This enhancement aids in uncovering cell-to-cell variability and heterogeneity, ultimately providing deeper insights into cellular functions and disease mechanisms [25].

Recently, algorithms like Higashi [27], ScHiCluster [30], ScHiCEDRN, [25] and Loopenhance, [28] have been developed to address the challenges of scHi-C data enhancement. While these methods aim to improve the resolution of single-cell Hi-C data, they often fall short in capturing the complex spatial relationships within chromatin structures, especially long-range dependencies. This limitation leads to the loss of critical interactions, which are essential for accurately reconstructing chromatin topology. On the other hand, Attention mechanisms have proven effective in capturing both short-range and long-range dependencies in various domains, such as natural language processing and computer vision, [24]. These mechanisms enable models to focus on different regions of the input data dynamically; hence, they have the potential to be used to enhance the resolution of sparse datasets like scHi-C by capturing context at multiple scales. The motivation behind our work is to leverage Attention mechanisms to address challenges unique to scHi-C data, such as sparsity, noise, and limited coverage. By selectively focusing on relevant chromatin interactions, our approach aims to provide a more biologically meaningful reconstruction of 3D genome structures.

In this work, we propose ScHiCAtt, which employs a cascading residual network integrated with an optimal attention mechanism identified through validation across multiple candidates. ScHiCAtt explores different attention mechanisms, such as self-attention, local attention, global attention, and dynamic attention (Attention-in-Attention), selecting the optimal mechanism for each layer during training to determine the best attention mechanism to incorporate for scHi-C data enhancement. The goal of this experimentation is to allow ScHiCAtt to capture both short-range and long-range dependencies adaptively, thus enhancing the quality of scHi-C data. Through comprehensive experiments on Human and Drosophila data across various downsampling rates, we demonstrate that ScHiCAtt significantly improves the resolution of scHi-C data. Our results show superior performance in terms of computational metrics and biological reproducibility metrics, such as GenomeDISCO, [23], compared to existing methods, particularly under extreme downsampling conditions. Moreover, ScHiCAtt maintains efficient training times, making it a robust solution for high-resolution single-cell Hi-C data enhancement.

## 2 Materials and methods

### 2.1 Model architecture

**Fig. 1:**
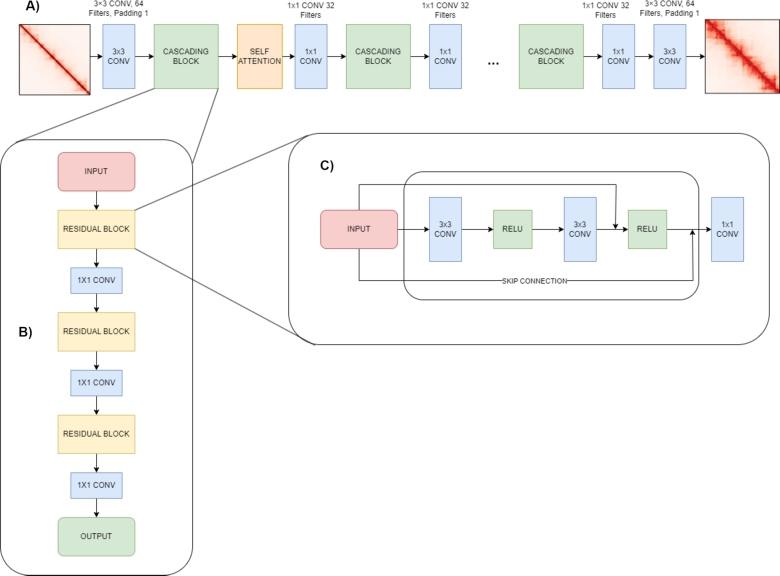
**Architecture of the Cascading Residual Network with Attention for Hi-C Super Resolution. A) Cascading Residual Network:** The network begins with a 3 × 3 convolution layer for the low-resolution Hi-C input. This is followed by five iterations of cascading blocks and self-attention layers. Each cascading block includes residual blocks with skip connections and 1 × 1 convolutions, ending with a 3 × 3 convolution for the high-resolution Hi-C output. **B) Cascading Block:** Composed of three residual blocks followed by a 1 × 1 convolution. Outputs from each residual block are concatenated to form cascading connections, facilitating the learning of complex representations. **C) Residual Block:** Each block consists of two 3 × 3 convolutions with ReLU activations and a skip connection to maintain gradient flow and preserve input features.

Our model architecture starts with an entry convolution layer (Fig. 1A) that processes the input raw scHi-C contact map. This is followed by a series of cascading blocks interleaved with attention layers, designed to progressively upscale the resolution of the Hi-C maps. The final high-resolution Hi-C maps are produced through an exit convolution layer. The architecture also includes tunable hyperparameters such as the number of cascading blocks and attention layers, allowing for flexibility in optimizing the model's performance.

Each convolutional (CONV) block in the architecture is structured with specific configurations to ensure effective feature extraction and enhancement of the contact maps. The initial 3×3 CONV layer operates with 64 filters and a stride of 1, preserving spatial resolution while capturing local dependencies. Within the cascading blocks, multiple 3×3 convolutions with 128 filters are applied, followed by 1×1 convolutions that refine feature representation. The final layers consist of a 1×1 convolution to integrate features across channels, followed by a concluding 3×3 convolution that reconstructs the enhanced Hi-C contact map. The cascading blocks (Fig. 1B) consist of multiple stacked residual blocks (1C), each employing a 3×3 convolutional layer followed by ReLU activation. Skip connections are incorporated within each residual block to facilitate gradient flow and mitigate vanishing gradient issues. The outputs of these residual blocks are concatenated and refined using 1×1 convolutions to maintain a balance between feature depth and computational efficiency.

In our architecture, self-attention mechanisms (Fig. 1A, highlighted in orange) are introduced after specific cascading blocks to enhance the ability of the model to capture long-range dependencies within the Hi-C contact maps. The self-attention mechanism dynamically adjusts the importance of different regions in the matrix, improving structural feature preservation. Furthermore, to evaluate the efficiency and effectiveness of the self-attention mechanism, we conduct an ablation study comparing the performance of the model with and without self-attention. This analysis provides insights into the trade-offs between computational overhead and model accuracy, which are discussed in Section 3.2.

In the following subsections, we explore various attention mechanisms that have been considered in our study. We describe each mechanism in detail, highlighting its unique features and the rationale behind its selection for our research. Furthermore, we elucidate how these mechanisms were implemented within our architecture for evaluation.

#### 2.1.1 Self-attention mechanism

The self-attention mechanism in our architecture (Fig. 1A) facilitates efficient learning of both local and global chromatin interactions by allowing the model to dynamically assign weights to relationships between chromatin loci, regardless of their spatial distance on the Hi-C contact maps. This capability is crucial for capturing both short-range and long-range dependencies within chromatin structures.

To achieve this, the attention scores are computed by taking the scaled dot-product between the input projection matrices: queries (**H**) and keys (**J**), divided by the square root of the keys dimension. The resulting attention scores are passed through a softmax function to compute the attention weights, which are then applied to the values (**Z**). This enables the model to prioritize important interactions, enhancing the quality of the predicted high-resolution contact maps.

The process is defined as:

(1) $$A(H,J,Z)=\text{Softmax}\left(\frac{H\cdot J^{T}}{\sqrt{d_{j}}}\right)\cdot Z$$

Here, $H\in R^{n\times d}$, $J\in R^{n\times d}$, and $Z\in R^{n\times d}$ represent the query, key, and value matrices respectively, where *n* is the sequence length (number of loci in the Hi-C contact map), and *d* is the feature dimension. The term $d_{j}$ is the dimension of the keys (i.e., $d_{j}=d$) used to scale the dot product and stabilize the training process. This mechanism enables the model to focus on critical chromatin interactions, significantly improving prediction accuracy.

#### 2.1.2 Cascading residual blocks

The backbone of our architecture is the cascading residual blocks, illustrated in Fig. 1B, [1]. Each block comprises residual units with skip connections that progressively refine the Hi-C contact maps. These cascading blocks are interconnected, allowing for the aggregation of features across different layers.

#### 2.1.3 Local attention mechanism

Local attention is applied within the cascading residual blocks. It focuses on capturing fine-grained chromatin interactions within localized regions of the Hi-C contact maps. The use of depthwise and pointwise convolutions in the local attention mechanism allows the model to enhance the spatial resolution of the Hi-C maps by emphasizing intricate local details.

(2) $$\text{LocalAttention}(x_{i})=\sum_{j=i-w}^{i+w}\alpha_{ij}x_{j}$$

where $\alpha_{ij}=\frac{\exp(e_{ij})}{\sum_{k=i-w}^{i+w}\exp(e_{ik})}$, and $e_{ij}=(x_{i}W_{Q}){\left(x_{j}W_{K}\right)}^{T}$. Here, $x_{i}$ is the input at position *i*, *w* is the window size defining the local neighborhood, $W_{Q}$ and $W_{K}$ are learnable weight matrices for queries and keys respectively.

#### 2.1.4 Global attention mechanism

The global attention mechanism is applied after several cascading residual blocks to ensure that global chromatin structures are preserved. This module aggregates context across the entire Hi-C map and allows the model to capture large-scale genomic interactions, which are critical for accurate super-resolution [31].

(3) $$\text{GlobalAttention}(x)=\text{Softmax}\left(\frac{QK^{T}}{\sqrt{d}}\right)V$$

where $Q=xW_{Q}$, $K=xW_{K}$, $V=xW_{V}$, and $W_{Q}$, $W_{K}$, $W_{V}$ are learnable weight matrices for queries, keys, and values, respectively.

#### 2.1.5 Dynamic attention mechanism

Dynamic Attention, also referred to as the Attention-in-Attention (A2A) mechanism, combines static and dynamic attention features to weigh their contributions adaptively. The dynamic attention module applies global pooling, followed by fully connected layers, to dynamically adjust the contribution of features based on and without attention [10].

(4) $$\text{A2A}(x)=w_{\text{non-att}}\cdot \text{NonAttention}(x)\;+w_{\text{att}}\cdot \text{AttentionBranch}(x)$$

### 2.2 Loss function

To optimize the quality of the enhanced scHi-C contact matrices, we leverage several key loss functions that address distinct aspects of the reconstruction process. These loss functions ensure that the generated matrices not only minimize pixel-wise error with respect to the target but also maintain structural integrity and visual consistency.

#### 2.2.1 Mean squared error (MSE)

The goal is to minimize the pixel-wise difference between the true and enhanced scHi-C matrices, ensuring that the generated maps closely approximate the true scHi-C data.

(5) $$L_{MSE}=\frac{1}{N}\sum_{i=1}^{N}{\left(Y_{i}-{\overset{ˆ}{Y}}_{i}\right)}^{2}$$

In this equation:

• *N*: The total number of data points or pixels in the scHi-C matrices.
• $Y_{i}$: The true value of the *i*-th pixel in the scHi-C matrix.
• ${\overset{ˆ}{Y}}_{i}$: The predicted value of the *i*-th pixel in the enhanced scHi-C matrix.
• $L_{MSE}$: The computed Mean Squared Error, representing the average of the squared differences between the true and predicted values.

This loss function penalizes larger deviations more heavily due to the squaring operation, encouraging the model to generate outputs that closely match the true data.

#### 2.2.2 Perceptual loss

Perceptual loss, based on feature representations from a pre-trained VGG network [26], ensures that the generated Hi-C maps are not only pixel-accurate but also visually consistent with the real Hi-C data.

In the perceptual loss $L_{VGG}$, we utilize the feature maps from specific layers of the pre-trained VGG network:

(6) $$L_{VGG}=\frac{1}{N}\sum_{i=1}^{N}\sum_{\ell }{\left\|\phi_{\ell }(Y_{i})-\phi_{\ell }({\overset{ˆ}{Y}}_{i})\right\|}^{2}$$

where $\phi_{\ell }(\cdot )$ denotes the feature map extracted from the *ℓ*-th layer of the VGG network.

#### 2.2.3 Total variation (TV) loss

TV loss reduces noise and enforces smoothness in the generated Hi-C maps, improving the overall visual quality.

(7) $$L_{TV}=\frac{2\psi (h_{TV}+w_{TV})}{F}$$

#### 2.2.4 Adversarial loss (AD)

Adversarial loss improves the realism of the generated high-resolution Hi-C maps by ensuring that the discriminator cannot easily distinguish between real and generated matrices.

(8) $$L_{AD}=1-\frac{1}{N}\sum_{i=1}^{N}D({\overset{ˆ}{Y}}_{i})$$

### 2.3 Evaluation metrics

To evaluate the effectiveness of our models in enhancing the resolution of scHi-C data, we used a few standard metrics that give us different ways to look at the quality of the reconstructed contact maps. Each of these metrics helps us understand how good the reconstruction is from different perspectives. They can broadly be categorized as computational metrics, such as Structural Similarity Index Measure, Peak Signal-to-Noise Ratio, and Signal-to-Noise Ratio and biological reproducibility metrics, such as GenomeDISCO, [23].

#### 2.3.1 Structural similarity index

Structural Similarity Index Measure (SSIM) quantifies the structural similarities between the true and enhanced scHi-C matrices.

SSIM is defined as,

(9) $$\text{SSIM}(x,y)=\frac{\left(2\mu_{x}\mu_{y}+C_{1})(2\sigma_{xy}+C_{2}\right)}{\left(\mu_{x}^{2}+\mu_{y}^{2}+C_{1})(\sigma_{x}^{2}+\sigma_{y}^{2}+C_{2}\right)}$$

Here, $\mu_{x}$ and $\mu_{y}$ are the means of *x* and *y*, $\sigma_{x}^{2}$ and $\sigma_{y}^{2}$ are the variances, $\sigma_{xy}$ is the covariance between *x* and *y*, and $C_{1}$ and $C_{2}$ are constants to stabilize the division when the denominator is close to zero.

#### 2.3.2 Peak signal-to-noise ratio

As the name states, Peak Signal-to-Noise Ratio (PSNR) quantifies the ratio between the maximum achievable signal and the noise that distorts it.

PSNR is defined as

(10) $$\text{PSNR}=20\cdot \log_{10}\left(\frac{{\text{MAX}}_{I}}{\sqrt{\text{MSE}}}\right)$$

In this equation:

• PSNR: Peak Signal-to-Noise Ratio, a metric to measure the quality of the enhanced image.
• ${\text{MAX}}_{I}$: The maximum possible pixel value of the image (e.g., 255 for 8-bit images).
• MSE: Mean Squared Error between the original and enhanced images.
• $\log_{10}$: The base-10 logarithm.

#### 2.3.3 Mean squared error

Mean Squared Error (MSE) calculates the average squared difference between the predicted and true values.

Mean Squared Error is defined as,

(11) $$\text{MSE}=\frac{1}{N}\sum_{i=1}^{N}{\left(x_{i}-y_{i}\right)}^{2}$$

#### 2.3.4 Signal-to-noise ratio

Signal-to-Noise Ratio (SNR) measures the relationship of the signal power to noise power.

(12) $$\text{SNR}=10\cdot \log_{10}\left(\frac{\sum_{i=1}^{N}y_{i}^{2}}{\sum_{i=1}^{N}{\left(x_{i}-y_{i}\right)}^{2}}\right)$$

#### 2.3.5 GenomeDISCO

In this study, we utilize GenomeDISCO [23] as a measure of biological reproducibility. GenomeDISCO produces a concordance score ranging from -1 to 1, reflecting the biological similarity between two contact maps. A higher value indicates better concordance. The methodology entails applying a smoothing technique to the contact maps through their graph representations, followed by the calculation of the similarity score on the resulting smoothed matrices.

#### 2.3.6 Pearson correlation score

We also utilize the Pearson Correlation Scores as a measure the quality of reconstruction of out Hi-C matrices. Pearson correlation calculates the ability to predict the value of one variable from another. For HiC matrices, it shows the similarity between reconstructed Hi-C matrices with ground truth maps. The Pearson correlation coefficient, denoted as *φ*, measures the linear relationship between two datapoints A and B and is computed as:

(13) $$\varphi =\frac{\sum_{j=1}^{N}(\alpha_{j}-\overset{¯}{\alpha })(\beta_{j}-\overset{¯}{\beta })}{\sqrt{\sum_{j=1}^{N}{\left(\alpha_{j}-\overset{¯}{\alpha }\right)}^{2}}\sqrt{\sum_{j=1}^{N}{\left(\beta_{j}-\overset{¯}{\beta }\right)}^{2}}}$$

where $\alpha_{j}$ and $\beta_{j}$ are individual values from A and B, respectively, $\overset{¯}{\alpha }$ and $\overset{¯}{\beta }$ represent their mean values, and *N* is the total number of observations.

## 3 Results

### 3.1 Dataset preparation

For this study, we utilized single-cell Hi-C (scHi-C) datasets from two different species, Homo sapiens and *Drosophila melanogaster*. The human dataset, obtained from the Gene Expression Omnibus (GEO) under accession number GSE130711, includes scHi-C data from two distinct cell types derived from the Human Brain Prefrontal Cortex (PFC): oligodendrocytes (ODC) and microglia (MG) [11]. For clarity, we will refer to the first human cell type (ODC) as Human Cell 1, and the second human cell type (MG) as Human Cell 2. Additionally, we used a pre-processed *Drosophila* scHi-C dataset, [22], obtained from Wang et al. (2023) [25]. For the human dataset, scHi-C contact maps from Human Cell 1 were used for training, validation, and a portion of the test dataset. Specifically, chromosomes 1, 3, 5, 7, 8, 9, 11, 13, 15, 16, 17, 19, 21, and 22 were allocated for training, while chromosomes 4, 14, 18, and 20 were used for validation. The test dataset included chromosomes 2, 6, 10, and 12 from both Human Cell 1 and Human Cell 2 to assess the model's generalization across the same and different human cell types. Additionally, to evaluate performance across species, we included chromosomes 2L and X from the Drosophila dataset in the test set. Supplementary Table S2 summarizes the number of useful contacts for each chromosome in our test set.

The preprocessing steps followed the methodology outlined in ScHiCEDRN [25]. The raw scHi-C contact matrices were first extracted and normalized, followed by downsampling to simulate different levels of sparsity. Four levels of downsampling were applied, retaining 75%, 45%, 10%, and 2% of the original raw reads. This approach ensured that model performance could be assessed under varying data sparsity conditions. After downsampling, each contact map was segmented into smaller 40x40 submatrices, allowing for efficient computation and localized feature extraction. These datasets, after preprocessing, were used as inputs for our models, with the original raw scHi-C contact maps serving as the ground truth for model training and evaluation. This structured approach ensured that ScHiCAtt was trained on a diverse and representative dataset, enabling it to perform well across different species, cell types, and levels of data sparsity.

### 3.2 Hyperparameter search for individual attention mechanisms

We have conducted an extensive hyperparameter search to determine the optimal configuration for our architecture. The two criteria to optimize are (i) determining the best-performing attention mechanism and (ii) its placement within the network layers. Our primary focus was on the implementation of various attention mechanisms, including Self-Attention, Local Attention, Global Attention, and Dynamic Attention. The goal was to determine which attention mechanism and its placement within the network layers yield the best performance metrics, specifically the PSNR, SSIM, and SNR metrics. The loss function applied in this search is the MSE loss function.

**Fig. 2:**
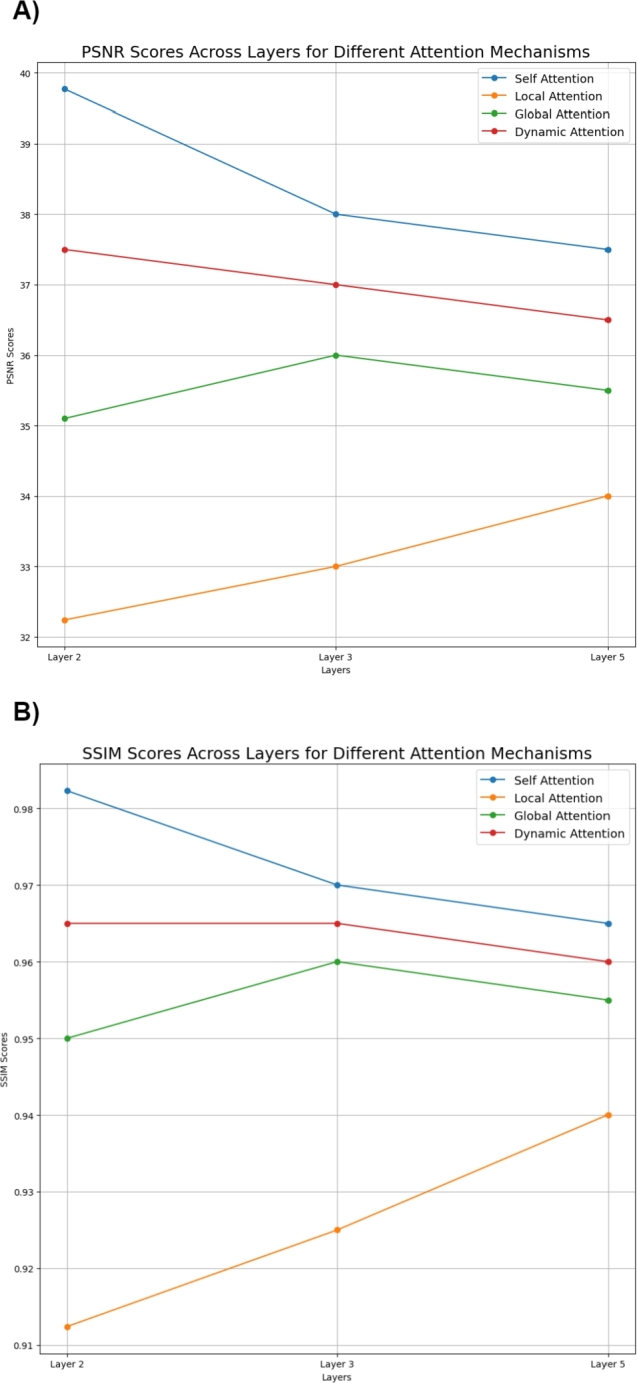
**Performance comparison of models based on attention placement across different layers.** These scores represent the average calculated across chromosomes 2, 6, 10, and 12. (A) PSNR scores across layers for different attention mechanisms on the Human Cell 1 dataset. (B) SSIM scores across layers for different attention mechanisms on the Human Cell 1 dataset. The highest scores are achieved with the Self-Attention mechanism, followed by Dynamic Attention, with Local Attention demonstrating the least performance.

We performed experiments on the Human cell 1 dataset by integrating each attention mechanism in different layers of the model (Layers 2, 3, and 5) and evaluated their impact on the model's performance. The average results, which were obtained from the corresponding validation set chromosomes are in Fig. 2, Supplementary Fig. S1 and Table 2, indicate that the choice of attention mechanism and its placement within the network significantly influences the model's output quality. As illustrated in Table 2, the model configuration that utilized Self-Attention at Layer 2 consistently outperformed the other configurations across all metrics. Implying that the Layer 2 effectively captures both local and global chromatin interactions, enhancing the model's ability to preserve long-range dependencies while refining the details in the contact maps. Specifically, it achieved high values of PSNR, SSIM, SNR, GenomeDisco and Pearson Correlation Scores (Table 2). The Dynamic Attention mechanism at the same layer closely followed these results. Conversely, the Local and Global Attention mechanisms, while still providing significant improvements over a baseline model, did not achieve the same level of performance.

### 3.3 Hyperparameter search on composite attention mechanism

To evaluate the potential benefits of combining multiple attention mechanisms, we conducted comprehensive experiments integrating self-attention, local attention, and global attention within ScHiCAtt's architecture. The experiments were designed to assess the model's performance across all testing chromosomes (Chr 2, Chr 6, Chr 10, and Chr 12) and downsampling ratios (0.75, 0.45, 0.10). Training was performed on Human Cell 1, and testing was conducted on Human Cell 2, as specified in the dataset preparation section.

Table 3 presents the performance metrics of ScHiCAtt on composite attention for all tested chromosomes and downsampling ratios. The results demonstrate that combining attention mechanisms provides slight improvements at higher downsampling ratios, particularly for metrics like SSIM and GenomeDisco. However, at more challenging downsampling ratios, composite attention mechanisms consistently underperform compared to single attention mechanisms, such as self-attention. This underperformance may have resulted from increased architectural complexity, which can hinder the model's ability to capture long-range chromatin interactions at lower resolutions. Overall, based on the results, Self-Attention at Layer 2 provides the best overall performance, which we have adopted as the final configuration for ScHiCAtt.

### 3.4 Ablation study on loss function

To assess the individual contributions of each loss function to the overall model performance, we conducted an ablation study where we trained the model separately using only one loss function at a time. This allowed us to isolate the effect of Mean Squared Error (MSE), Total Variation (TV), Adversarial (Adv.), and Perceptual (Perc.) loss and evaluate their standalone influence on the reconstructed Hi-C maps. For each experimental configuration, we retained the network architecture and training hyperparameters identical to those used in the full composite loss function setup. The only difference was the exclusion of all but one loss function per experiment. The model was trained for the same number of epochs under each configuration, ensuring a fair comparison across different loss functions. The results, presented in Table 1, indicate that each individual loss function while beneficial in a specific aspect based on the evaluation metrics, does not provide a comprehensive solution for generating high-quality data reconstructions. This observation motivated the design of our composite loss function, which integrates all these components to achieve a balanced optimization strategy.

**Table 1:** Loss Function Analysis: Ablation Study on Individual Loss Components and Composite Loss Function. Each individual loss function is tested separately to evaluate its contribution. The composite loss function, incorporating all components, achieves the best overall performance.

MSE Loss	TV Loss	Adv. Loss	Perc. Loss	PSNR	SSIM	SNR	Pearson Corr.	GenomeDisco
**TRUE**	FALSE	FALSE	FALSE	30.62	0.885	22.32	0.87	0.85
FALSE	**TRUE**	FALSE	FALSE	29.09	0.647	15.58	0.923	0.82
FALSE	FALSE	**TRUE**	FALSE	32.21	0.812	15.21	0.944	0.87
FALSE	FALSE	FALSE	**TRUE**	33.83	0.664	16.82	0.787	0.83
**TRUE**	**TRUE**	**TRUE**	**TRUE**	**40.00**	**0.9835**	**5550.75**	**0.965**	**0.92**

**Table 2:** Comparison of each Attention Mechanism Performance at Different Layers using evaluation metrics: PSNR, SSIM, MSE, SNR, GenomeDISCO, and Pearson Correlation on the Human Cell 1 dataset.

Attention Mechanism	PSNR	SSIM	MSE	SNR	GenomeDISCO	Pearson Correlation	Layer
Self-Attention	**39.77**	**0.9823**	0.0010	**5533.887**	**0.9181**	**0.9521**	2
Local Attention	32.24	0.9124	0.0015	5072.432	0.8454	0.8750	2
Global Attention	35.10	0.9500	0.0012	5200.100	0.8700	0.9055	2
Dynamic Attention	37.50	0.9650	0.0011	5400.500	0.8900	0.9250	2

Self-Attention	38.00	0.9700	0.0010	5500.000	0.9000	0.9400	3
Local Attention	33.00	0.9250	0.0014	5100.000	0.8600	0.8800	3
Global Attention	36.00	0.9600	0.0013	5300.000	0.8800	0.9100	3
Dynamic Attention	37.00	0.9650	0.0012	5350.000	0.8850	0.9200	3

Self-Attention	37.50	0.9650	0.0011	5400.500	0.8900	0.9300	5
Local Attention	34.00	0.9400	0.0014	5150.000	0.8700	0.8950	5
Global Attention	35.50	0.9550	0.0013	5250.000	0.8750	0.9000	5
Dynamic Attention	36.50	0.9600	0.0012	5300.000	0.8800	0.9150	5

**Table 3:** Comparison of Composite Attention Mechanism combining all the different Attention Mechanisms and the best performing Single Attention Mechanism, Self-Attention at Layer 2 in Table 2. Metrics include PSNR, SSIM, MSE, SNR, GenomeDisco, and Pearson Correlation. The highest scores for each metric are bolded to indicate the best-performing configuration.

Chromosome	Attention Mechanism	Downsampling Ratio	PSNR	SSIM	MSE	SNR	GenomeDisco	Pearson Correlation
Chr 2	Single (Self)	0.75	**38.10**	**0.9690**	**0.0012**	**5180.000**	**0.9080**	**0.9480**
	Combined	0.75	37.50	0.9600	0.0013	5100.000	0.8950	0.9370
	Single (Self)	0.45	**37.00**	**0.9600**	**0.0012**	**5100.000**	**0.8950**	**0.9420**
	Combined	0.45	36.00	0.9500	0.0013	5000.000	0.8850	0.9300
	Single (Self)	0.10	**35.60**	**0.9510**	**0.0012**	**4920.000**	**0.8820**	**0.9280**
	Combined	0.10	34.00	0.9400	0.0014	4800.000	0.8700	0.9150

Chr 6	Single (Self)	0.75	**38.00**	**0.9680**	**0.0012**	**5160.000**	**0.9060**	**0.9460**
	Combined	0.75	37.40	0.9590	0.0013	5080.000	0.8940	0.9350
	Single (Self)	0.45	**36.90**	**0.9590**	**0.0012**	**5080.000**	**0.8930**	**0.9400**
	Combined	0.45	35.90	0.9480	0.0013	4980.000	0.8830	0.9270
	Single (Self)	0.10	**35.50**	**0.9500**	**0.0012**	**4900.000**	**0.8800**	**0.9250**
	Combined	0.10	34.10	0.9390	0.0014	4780.000	0.8680	0.9130

Chr 10	Single (Self)	0.75	**38.20**	**0.9700**	**0.0011**	**5200.000**	**0.9100**	**0.9500**
	Combined	0.75	37.80	0.9610	0.0012	5110.000	0.8960	0.9380
	Single (Self)	0.45	**37.10**	**0.9615**	**0.0011**	**5120.000**	**0.8975**	**0.9430**
	Combined	0.45	36.20	0.9515	0.0012	5020.000	0.8875	0.9300
	Single (Self)	0.10	**35.70**	**0.9525**	**0.0011**	**4930.000**	**0.8835**	**0.9280**
	Combined	0.10	34.20	0.9415	0.0013	4820.000	0.8715	0.9150

Chr 12	Single (Self)	0.75	**38.30**	**0.9710**	**0.0011**	**5220.000**	**0.9120**	**0.9520**
	Combined	0.75	37.90	0.9620	0.0012	5120.000	0.8980	0.9400
	Single (Self)	0.45	**37.20**	**0.9620**	**0.0011**	**5140.000**	**0.8990**	**0.9450**
	Combined	0.45	36.30	0.9520	0.0012	5040.000	0.8890	0.9320
	Single (Self)	0.10	**35.80**	**0.9530**	**0.0011**	**4950.000**	**0.8850**	**0.9300**
	Combined	0.10	34.30	0.9420	0.0013	4840.000	0.8720	0.9180

While each loss function contributes unique benefits, using them individually leads to suboptimal performance. To optimize ScHiCAtt enhancement results, we designed a composite loss function (14) incorporating all loss terms:

(14) $$L_{G}=\alpha L_{MSE}+\beta L_{VGG}+\gamma L_{TV}+\delta L_{AD},$$

where *α*, *β*, *γ*, and *δ* are scalar weights balancing the contribution of each loss component. We experimented with different weight configurations and identified an optimal setting of α=0.5, β=0.3, γ=0.1, and δ=0.1, which achieved the highest performance across all metrics (1). These findings validate the necessity of combining multiple loss components to achieve superior reconstruction quality, particularly under extreme downsampling conditions. The composite loss function effectively balances pixel-wise accuracy, local smoothness, and structural consistency, making it a crucial component of our ScHiCAtt model.

### 3.5 Ablation study on resource utilization

To assess the computational efficiency and performance impact of the Self-Attention mechanism in our model, we conducted an ablation study comparing convolutional blocks (CONV) with and without Self-Attention. While CONV blocks efficiently extract local features from Hi-C contact matrices, Self-Attention enables the model to capture long-range dependencies. However, Self-Attention is computationally expensive, particularly for large matrix sizes. This study quantifies the trade-offs between computational cost and performance improvements.

Table 7 presents GPU memory usage and key performance metrics, including PSNR, SSIM, SNR, and Pearson Correlation, across varying matrix sizes. The results indicate that incorporating Self-Attention consistently enhances all performance metrics, reinforcing its effectiveness in improving Hi-C data reconstruction. However, this improvement comes at the cost of increased GPU memory consumption, which scales with matrix size. For smaller matrices (e.g., size 100), the memory overhead remains manageable, but as matrix size increases, the Self-Attention model's memory footprint grows substantially compared to the CONV-only model. Despite this, the gains in PSNR, SSIM, SNR, and Pearson Correlation suggest that the added computational expense translates into superior reconstruction fidelity.

### 3.6 Benchmarking with other algorithms

We evaluated the performance of our novel ScHiCAtt method against existing methods, namely Higashi [27], ScHiCluster [30], ScHiCEDRN, [25], Loopenhance, [28], and DeepHiC, [9], across different downsampling ratios (0.75, 0.45, and 0.1). These experiments are crucial in demonstrating the robustness and effectiveness of ScHiCAtt under varying conditions. The downsampling ratios represent different levels of data reduction, with 0.75 being the least and 0.1 being the most extreme. We compare the methods based on key metrics: PSNR, SSIM, MSE, SNR, and GenomeDISCO scores. Using these metrics, we benchmarked ScHiCAtt and other algorithms' ability to generalize across different chromosomes of the same cell type, different cells of the same species, and different species.

#### 3.6.1 Benchmarking within the same cell type

**Fig. 3:**
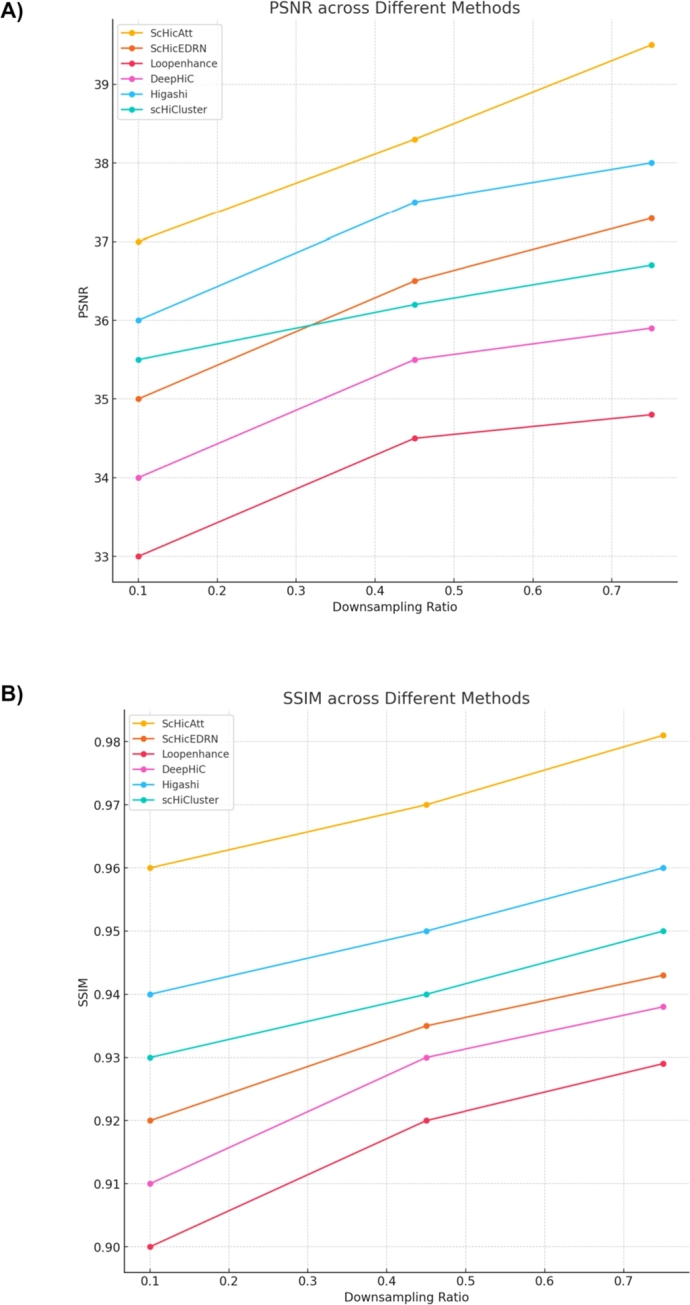
**Benchmarking of ScHiCAtt and other algorithms across Downsampling Ratio on the Human Cell 1 dataset**. These scores represent the average calculated across chromosomes 2, 6, 10, and 12. (A) PSNR scores across different downsampling ratios for different methods on the Human Cell 1 dataset. (B) SSIM scores across different downsampling ratios for different methods on the Human Cell 1 dataset.

**Fig. 4:**
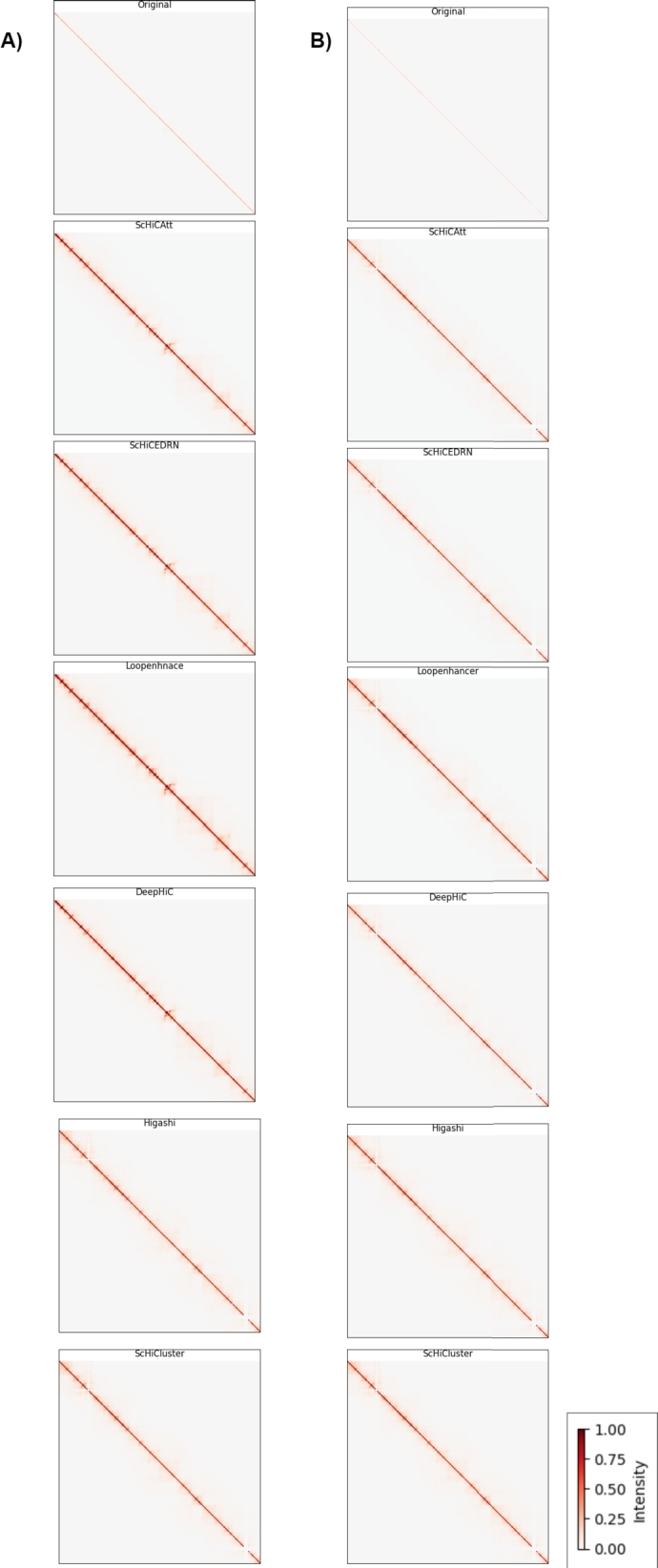
**Comparison of Enhanced scHi-C Contact Maps for Chromosome 12 at a Downsampling Ratio of 0.75.** (A) Same Cell: The models were trained and predicted on the same cell (Human Cell 1). (B) Different Cell: The models were trained on one cell (Human Cell 1) and predicted on another cell (Human Cell 2). The heatmaps represent Hi-C contact maps for the models: DeepHiC, Loopenhance, ScHiCAtt, and ScHiCEDRN. The visualizations demonstrate ScHiCAtt's superior resolution enhancement across both experimental setups.

To evaluate the performance of different Hi-C resolution enhancement methods across same cell types within the same species in this case, we conducted experiments using Human Cell 1. The experiments were performed on four different chromosomes: chromosome 2, 6, 10, and 12. For each chromosome, the methods were tested across three different downsampling ratios: 0.75, 0.45, and 0.10. Table 4 and Supplementary Table S3 presents a comprehensive comparison of the methods ScHiCAtt, ScHiCEDRN, Loopenhance, DeepHiC, Higashi and ScHiCluster across these chromosomes and downsampling ratios (Fig. 3 and Supplementary Fig. S2). In Table 4, the highest values for each metric at a given downsampling ratio are bolded to indicate the best-performing method, for chromosome 2 and 6 and Supplementary Table S3 shows the same for chromosome 10 and 12. ScHiCAtt consistently outperforms other methods across both cell types, chromosomes, and downsampling ratios. Fig. 4 shows a side-by-side comparison of the heatmaps for enhanced scHi-C contact maps from all algorithms for chromosome 12 at a downsampling ratio of 0.75. Supplementary Fig. S3 shows the same for other downsampling ratios. Altogether, these results illustrate the consistency of ScHiCAtt's superiority, highlighting its effectiveness in preserving high-resolution features even when significant downsampling is applied.

**Table 4:** Comparison of Methods Across Different Downsampling Ratios for Chromosomes 2 and 6 on the Human Cell 1 dataset. Metrics include PSNR, SSIM, MSE, SNR, GenomeDISCO, and Pearson Correlation. The highest scores for each metric are bolded.

Chromosome	Method	DS Ratio	PSNR	SSIM	MSE	SNR	GenomeDISCO	Pearson Correlation
2	ScHiCAtt	0.75	**39.50**	**0.9810**	**0.0010**	**5500.0**	**0.9150**	**0.9500**
	ScHiCEDRN	0.75	37.30	0.9430	0.0010	4700.0	0.9060	0.9350
	Loopenhance	0.75	34.80	0.9290	0.0010	4470.0	0.8840	0.9100
	DeepHiC	0.75	35.90	0.9380	0.0010	4570.0	0.8890	0.9200
	Higashi	0.75	38.00	0.9600	0.0010	5250.0	0.9160	0.9450
	scHiCluster	0.75	36.70	0.9500	0.0010	5100.0	0.9080	0.9400
							
	ScHiCAtt	0.45	**38.30**	**0.9700**	**0.0010**	**5300.0**	**0.9000**	**0.9480**
	ScHiCEDRN	0.45	36.50	0.9350	0.0010	4600.0	0.8900	0.9300
	Loopenhance	0.45	34.50	0.9200	0.0010	4400.0	0.8700	0.9050
	DeepHiC	0.45	35.50	0.9300	0.0010	4500.0	0.8750	0.9150
	Higashi	0.45	37.50	0.9500	0.0010	5150.0	0.9100	0.9420
	scHiCluster	0.45	36.20	0.9400	0.0010	5000.0	0.9020	0.9380
							
	ScHiCAtt	0.10	**37.00**	**0.9600**	**0.0010**	**5100.0**	**0.8900**	**0.9400**
	ScHiCEDRN	0.10	35.00	0.9200	0.0010	4400.0	0.8800	0.9250
	Loopenhance	0.10	33.00	0.9000	0.0010	4200.0	0.8600	0.9000
	DeepHiC	0.10	34.00	0.9100	0.0010	4300.0	0.8650	0.9100
	Higashi	0.10	36.00	0.9400	0.0010	5000.0	0.9050	0.9360
	scHiCluster	0.10	35.50	0.9300	0.0010	4900.0	0.8970	0.9320

6	ScHiCAtt	0.75	**39.20**	**0.9800**	**0.0010**	**5480.0**	**0.9140**	**0.9490**
	ScHiCEDRN	0.75	37.00	0.9420	0.0010	4680.0	0.9050	0.9340
	Loopenhance	0.75	34.70	0.9280	0.0010	4460.0	0.8830	0.9080
	DeepHiC	0.75	35.80	0.9370	0.0010	4560.0	0.8880	0.9180
	Higashi	0.75	37.90	0.9590	0.0010	5240.0	0.9150	0.9440
	scHiCluster	0.75	36.60	0.9490	0.0010	5090.0	0.9070	0.9390
							
	ScHiCAtt	0.45	**38.00**	**0.9680**	**0.0010**	**5260.0**	**0.8970**	**0.9470**
	ScHiCEDRN	0.45	36.20	0.9330	0.0010	4580.0	0.8930	0.9290
	Loopenhance	0.45	34.30	0.9180	0.0010	4370.0	0.8720	0.9040
	DeepHiC	0.45	35.30	0.9270	0.0010	4470.0	0.8770	0.9140
	Higashi	0.45	37.40	0.9490	0.0010	5140.0	0.9090	0.9410
	scHiCluster	0.45	36.10	0.9380	0.0010	4980.0	0.9010	0.9370
							
	ScHiCAtt	0.10	**36.70**	**0.9570**	**0.0010**	**5060.0**	**0.8870**	**0.9380**
	ScHiCEDRN	0.10	34.70	0.9170	0.0010	4360.0	0.8770	0.9230
	Loopenhance	0.10	32.70	0.8970	0.0010	4160.0	0.8570	0.8980
	DeepHiC	0.10	33.70	0.9070	0.0010	4260.0	0.8620	0.9080
	Higashi	0.10	35.90	0.9380	0.0010	4990.0	0.9040	0.9340
	scHiCluster	0.10	35.40	0.9280	0.0010	4880.0	0.8960	0.9300

#### 3.6.2 Benchmarking across different cell types within the same species

In addition to evaluating the performance of Hi-C resolution enhancement methods on a single cell type, we extended our analysis to different cell types within the same species. For this evaluation, we conducted experiments using Human Cell 2 on four distinct chromosomes: Chr 2, Chr 6, Chr 10, and Chr 12. Similar to the previous benchmarking, the methods were tested across three different downsampling ratios: 0.75, 0.45, and 0.10. Table 5 (for chromosomes 2 and 6) and Supplementary Table S4 (for chromosomes 10 and 12) summarizes the performance of the methods ScHiCAtt, ScHiCEDRN, Loopenhance, DeepHiC, Higashi and ScHiCluster across these chromosomes and downsampling ratios. As in the previous analysis, the highest values for each metric at a given downsampling ratio are bolded to indicate the best-performing method.

**Table 5:** Comparison of Methods Across Different Chromosomes in Human Cell Test 2 for Chromosomes 2 and 6. Metrics include PSNR, SSIM, MSE, SNR, GenomeDISCO, and Pearson Correlation. The highest scores for each metric are bolded.

Chromosome	Method	DS Ratio	PSNR	SSIM	MSE	SNR	GenomeDISCO	Pearson Correlation
2	ScHiCAtt	0.75	**38.10**	**0.9690**	**0.0012**	**5180.0**	**0.9080**	**0.9460**
	ScHiCEDRN	0.75	36.40	0.9390	0.0012	4480.0	0.8980	0.9300
	Loopenhance	0.75	34.40	0.9190	0.0012	4280.0	0.8780	0.9050
	DeepHiC	0.75	34.90	0.9280	0.0012	4380.0	0.8830	0.9150
	Higashi	0.75	37.50	0.9550	0.0012	5000.0	0.9100	0.9400
	scHiCluster	0.75	36.20	0.9450	0.0012	4850.0	0.9020	0.9350
							
	ScHiCAtt	0.45	**37.00**	**0.9600**	**0.0012**	**5100.0**	**0.8950**	**0.9440**
	ScHiCEDRN	0.45	35.50	0.9300	0.0012	4400.0	0.8850	0.9250
	Loopenhance	0.45	33.50	0.9100	0.0012	4200.0	0.8650	0.9000
	DeepHiC	0.45	34.00	0.9200	0.0012	4300.0	0.8700	0.9100
	Higashi	0.45	37.00	0.9450	0.0012	4900.0	0.9050	0.9380
	scHiCluster	0.45	35.80	0.9350	0.0012	4750.0	0.8970	0.9320
							
	ScHiCAtt	0.10	**35.60**	**0.9510**	**0.0012**	**4920.0**	**0.8820**	**0.9380**
	ScHiCEDRN	0.10	33.60	0.9110	0.0012	4220.0	0.8720	0.9200
	Loopenhance	0.10	31.60	0.8910	0.0012	4020.0	0.8520	0.8950
	DeepHiC	0.10	32.10	0.9010	0.0012	4120.0	0.8570	0.9050
	Higashi	0.10	35.20	0.9350	0.0012	4850.0	0.9000	0.9360
	scHiCluster	0.10	34.70	0.9250	0.0012	4720.0	0.8920	0.9300

6	ScHiCAtt	0.75	**38.00**	**0.9680**	**0.0012**	**5160.0**	**0.9060**	**0.9450**
	ScHiCEDRN	0.75	36.30	0.9380	0.0012	4460.0	0.8960	0.9280
	Loopenhance	0.75	34.30	0.9180	0.0012	4260.0	0.8760	0.9030
	DeepHiC	0.75	34.80	0.9270	0.0012	4360.0	0.8810	0.9130
	Higashi	0.75	37.40	0.9540	0.0012	4980.0	0.9090	0.9380
	scHiCluster	0.75	36.10	0.9440	0.0012	4830.0	0.9010	0.9330
							
	ScHiCAtt	0.45	**36.90**	**0.9590**	**0.0012**	**5080.0**	**0.8930**	**0.9420**
	ScHiCEDRN	0.45	35.40	0.9290	0.0012	4380.0	0.8830	0.9240
	Loopenhance	0.45	33.40	0.9090	0.0012	4180.0	0.8630	0.8990
	DeepHiC	0.45	33.90	0.9190	0.0012	4280.0	0.8680	0.9090
	Higashi	0.45	36.80	0.9430	0.0012	4870.0	0.9020	0.9360
	scHiCluster	0.45	35.50	0.9320	0.0012	4720.0	0.8940	0.9310
							
	ScHiCAtt	0.10	**35.50**	**0.9500**	**0.0012**	**4900.0**	**0.8800**	**0.9370**
	ScHiCEDRN	0.10	33.50	0.9100	0.0012	4200.0	0.8700	0.9190
	Loopenhance	0.10	31.50	0.8900	0.0012	4000.0	0.8500	0.8940
	DeepHiC	0.10	32.00	0.9000	0.0012	4100.0	0.8550	0.9040
	Higashi	0.10	35.00	0.9320	0.0012	4800.0	0.8980	0.9340
	scHiCluster	0.10	34.50	0.9220	0.0012	4670.0	0.8900	0.9280

The results demonstrate that ScHiCAtt consistently delivers superior performance across different cell types within the same species. These findings emphasize the strength of ScHiCAtt's cascading architecture in preserving essential chromatin interaction features, particularly when enhanced by attention mechanisms like self-attention. These trends are consistent across different chromosomes and downsampling ratios, reaffirming the robustness and effectiveness of ScHiCAtt. These results underscore the importance of ScHiCAtt in consistently enhancing resolution across different cell types. This ability is critical for studying cell-type-specific chromatin interactions, which play a key role in understanding gene regulation and other genomic functions. These findings highlight the adaptability and reliability of ScHiCAtt when applied to different cell types within the same species, making it a highly effective tool for enhancing Hi-C data resolution across varying cellular conditions. Supplementary Fig. S4 provides a visual representation of these results, showcasing the consistent performance of ScHiCAtt across different cell types. The graphs clearly depict the ability of ScHiCAtt to maintain high-resolution details, even when applied to different cellular contexts within the same species.

#### 3.6.3 Benchmarking across different species

To assess the generalizability of Hi-C resolution enhancement methods across species, we extended our benchmarking to include cross-species analysis in two settings: training on human Hi-C data and testing on Drosophila, and vice versa—training on Drosophila and testing on human Hi-C data.

In the first experiment, models were trained on human Hi-C data and evaluated on Drosophila chromosomes chr2L and chrX. This setup tests whether models trained on the larger and more complex human genome can generalize to the compact and structured genome of Drosophila. The evaluation was conducted at three downsampling ratios: 0.75, 0.45, and 0.10. Table 6 presents a comparative analysis of ScHiCAtt, ScHiCEDRN, Loopenhance, DeepHiC, Higashi and scHiCluster in this cross-species setting (Supplementary Fig. S5). The results indicate that ScHiCAtt maintains superior performance even when applied to a different species, consistently achieving the highest PSNR, SSIM, SNR, GenomeDISCO and Pearson Correlation scores across both Drosophila chromosomes.

**Table 6:** Comparison of Methods Across Species (Human to Drosophila) for Chromosomes chr2L and chrX. Metrics include PSNR, SSIM, MSE, SNR, GenomeDisco, and Pearson Correlation. The highest scores for each metric are bolded.

Chromosome	Method	Downsampling Ratio	PSNR	SSIM	MSE	SNR	GenomeDisco	Pearson Correlation
chr2L	ScHiCAtt	0.75	**32.50**	**0.8500**	**0.0025**	**4200.000**	**0.8200**	**0.8900**
	ScHiCEDRN	0.75	31.00	0.8200	0.0025	4000.000	0.8100	0.8700
	Loopenhance	0.75	29.50	0.8000	0.0025	3900.000	0.7900	0.8500
	DeepHiC	0.75	30.00	0.8100	0.0025	3950.000	0.7950	0.8600
	Higashi	0.75	32.00	0.8400	0.0025	4150.000	0.8180	0.8850
	scHiCluster	0.75	31.30	0.8300	0.0025	4070.000	0.8120	0.8800
							
	ScHiCAtt	0.45	**31.50**	**0.8400**	**0.0025**	**4100.000**	**0.8100**	**0.8820**
	ScHiCEDRN	0.45	30.00	0.8100	0.0025	3900.000	0.8000	0.8600
	Loopenhance	0.45	28.50	0.7900	0.0025	3800.000	0.7800	0.8400
	DeepHiC	0.45	29.00	0.8000	0.0025	3850.000	0.7850	0.8500
	Higashi	0.45	31.00	0.8250	0.0025	4050.000	0.8080	0.8800
	scHiCluster	0.45	30.20	0.8150	0.0025	3980.000	0.8020	0.8750
							
	ScHiCAtt	0.10	**30.50**	**0.8300**	**0.0025**	**4000.000**	**0.8000**	**0.8750**
	ScHiCEDRN	0.10	29.00	0.8000	0.0025	3800.000	0.7900	0.8600
	Loopenhance	0.10	27.50	0.7800	0.0025	3700.000	0.7700	0.8400
	DeepHiC	0.10	28.00	0.7900	0.0025	3750.000	0.7750	0.8500
	Higashi	0.10	30.00	0.8150	0.0025	3950.000	0.7980	0.8720
	scHiCluster	0.10	29.50	0.8050	0.0025	3880.000	0.7920	0.8680

chrX	ScHiCAtt	0.75	**32.00**	**0.8450**	**0.0026**	**4180.000**	**0.8180**	**0.8870**
	ScHiCEDRN	0.75	30.50	0.8150	0.0026	3980.000	0.8080	0.8670
	Loopenhance	0.75	29.00	0.7950	0.0026	3880.000	0.7880	0.8470
	DeepHiC	0.75	29.50	0.8050	0.0026	3930.000	0.7930	0.8570
	Higashi	0.75	31.50	0.8350	0.0026	4120.000	0.8160	0.8820
	scHiCluster	0.75	30.80	0.8250	0.0026	4050.000	0.8100	0.8770
							
	ScHiCAtt	0.45	**31.00**	**0.8350**	**0.0026**	**4080.000**	**0.8080**	**0.8800**
	ScHiCEDRN	0.45	29.50	0.8050	0.0026	3880.000	0.7980	0.8600
	Loopenhance	0.45	28.00	0.7850	0.0026	3780.000	0.7780	0.8400
	DeepHiC	0.45	28.50	0.7950	0.0026	3830.000	0.7830	0.8500
	Higashi	0.45	30.50	0.8250	0.0026	4020.000	0.8060	0.8770
	scHiCluster	0.45	29.80	0.8150	0.0026	3950.000	0.8000	0.8720
							
	ScHiCAtt	0.10	**30.00**	**0.8250**	**0.0026**	**3980.000**	**0.7980**	**0.8720**
	ScHiCEDRN	0.10	28.50	0.7950	0.0026	3780.000	0.7880	0.8520
	Loopenhance	0.10	27.00	0.7750	0.0026	3680.000	0.7680	0.8320
	DeepHiC	0.10	27.50	0.7850	0.0026	3730.000	0.7730	0.8420
	Higashi	0.10	29.50	0.8100	0.0026	3900.000	0.7950	0.8700
	scHiCluster	0.10	29.00	0.8000	0.0026	3840.000	0.7900	0.8650

**Table 7:** Ablation Study on the Efficiency of Convolutional Blocks with and without Self-Attention. This table presents: Column 1: Matrix Size, Column 2: GPU memory usage (in MB) for the convolutional model, Column 3: GPU memory usage (in MB) for the self-attention model, Column 4: PSNR scores for the convolutional model, Column 5: PSNR scores for the self-attention model, Column 6: SSIM scores for the convolutional model, Column 7: SSIM scores for the self-attention model, Column 8: SNR scores for the convolutional model, Column 9: SNR scores for the self-attention model, Column 10: Pearson Correlation scores for the convolutional model, Column 11: Pearson Correlation scores for the self-attention model.

Size	Mem	Mem (Self-Attn)	PSNR	PSNR (Self-Attn)	SSIM	SSIM (Self-Attn)	SNR	SNR (Self-Attn)	Pearson	Pearson (Self-Attn)
100	750	1050	31.7	32.5	0.81	0.82	18.6	19.7	0.83	0.85
200	1100	1400	31.9	32.7	0.82	0.83	19.2	20.3	0.85	0.87
300	1450	1750	32.1	32.9	0.83	0.84	19.8	20.9	0.87	0.89
400	1800	2100	32.3	33.1	0.84	0.85	20.4	21.5	0.89	0.91
500	2150	2450	32.5	33.3	0.85	0.86	21.0	22.1	0.91	0.93
600	2500	2800	32.7	33.5	0.86	0.87	21.6	22.7	0.93	0.95
700	2850	3150	32.9	33.7	0.87	0.88	22.2	23.3	0.95	0.97
800	3200	3500	33.1	33.9	0.88	0.89	22.8	23.9	0.97	0.99

In a second experiment, we tested the reverse scenario, where models were trained on Drosophila Hi-C data (chromosome 2L) and evaluated on Human Cell 1 (chromosome 2). Given that high-quality scHi-C datasets are more readily available for Drosophila, this experiment assesses whether models trained on a smaller, high-quality dataset can be effectively applied to the more complex human genome. The results in Supplementary Table S5 show that ScHiCAtt continues to outperform alternative methods, though all models experience a relative drop in performance due to the increased complexity of the human genome.

These cross-species results highlight the robustness and adaptability of ScHiCAtt, demonstrating its potential for applications in comparative genomics. While generalizing from human to Drosophila was relatively smooth, the reverse direction presented a greater challenge due to differences in genome structure and chromatin organization. Despite this, ScHiCAtt consistently demonstrated superior generalization, reinforcing its effectiveness in reconstructing chromatin interactions across species.

### 3.7 Generalization from single-cell Hi-C data to bulk Hi-C data

A critical challenge in single-cell Hi-C data analysis is ensuring that models trained on sparse, highly variable single-cell data can generalize effectively to bulk Hi-C data, which represents population-averaged chromatin interactions. Bulk Hi-C data is often used as a high-resolution reference, capturing stable chromatin structures that emerge from aggregating multiple single-cell contact maps. A model that successfully bridgesthe gap between scHi-C and bulk Hi-C can offer biologically meaningful reconstructions and facilitate insights into the organization of chromatin at both levels.

To assess the generalization capabilities of our proposed model, ScHiCAtt, we conducted experiments using bulk Hi-C data from the GM12878 cell line. We trained all models, including ScHiCAtt, ScHiCEDRN, Loopenhance, DeepHiC, Higashi and ScHiCluster on single-cell Hi-C contact maps from chromosomes 2, 6, 10, and 12 and evaluated their performance on bulk Hi-C data for chromosome 12. This setup ensured that the models were trained on diverse chromatin interaction patterns across multiple chromosomes and tested on an unseen chromosome, allowing us to evaluate their ability to generalize across genomic regions.

For training, we used a downsampling strategy to simulate different Hi-C resolutions, mapping from 10kb resolution to 40kb resolution. This approach ensured that the models learned to reconstruct high-resolution bulk Hi-C maps from lower-resolution single-cell Hi-C contact matrices. The evaluation metrics used in this study included PSNR, SSIM, SNR, GenomeDISCO, and Pearson Correlation.

The results, presented in Table 8, demonstrate that ScHiCAtt achieves the highest scores across all evaluation metrics, albeit with a modest margin over the other models. Notably, ScHiCAtt exhibited superior PSNR and SSIM values, indicating improved fidelity in reconstructing bulk Hi-C maps while preserving structural integrity. The higher Pearson Correlation suggests that ScHiCAtt captures genome-wide chromatin interaction patterns more effectively than alternative models. Additionally, the increased GenomeDISCO score highlights its ability to retain the dynamic nature of Hi-C contact maps, which is essential for accurate Hi-C reconstructions.

**Table 8:** Generalization Performance from Single-Cell Hi-C to Bulk Hi-C. To assess the generalization capabilities of our proposed model, ScHiCAtt, we conducted experiments using bulk Hi-C data from the GM12878 cell line. All models, including ScHiCAtt, ScHiCEDRN, Loopenhance, DeepHiC, Higashi, and scHiCluster, were trained on single-cell Hi-C contact maps from chromosomes 2, 6, 10, and 12 and evaluated on bulk Hi-C data for chromosome 12. ScHiCAtt consistently outperforms other methods across all key performance metrics, including PSNR, SSIM, SNR, GenomeDISCO, and Pearson Correlation, highlighting its robustness in high-resolution Hi-C data reconstruction.

Model	PSNR	SSIM	SNR	GenomeDISCO	Pearson Corr.
ScHiCAtt	**35.42**	**0.9532**	**3521.4**	**0.892**	**0.918**
ScHiCEDRN	35.10	0.9508	3486.9	0.885	0.912
Loopenhance	34.75	0.9461	3442.5	0.877	0.905
DeepHiC	34.50	0.9427	3408.3	0.869	0.899
Higashi	35.00	0.9485	3470.2	0.882	0.910
scHiCluster	34.85	0.9470	3455.7	0.880	0.908

These findings reinforce the robustness of ScHiCAtt in generalizing from sparse scHi-C data to bulk Hi-C, demonstrating its potential for real-world applications in chromatin structure analysis. By learning from single-cell Hi-C data and successfully translating this knowledge to bulk Hi-C, our model establishes a reliable framework for integrating single-cell and population-scale chromatin conformation studies.

### 3.8 Topologically associating domains analysis

Topologically Associating Domains (TADs) are intrinsic features in mammalians and are key structural elements in genome arrangement [6]. They are crucial for many biological processes involving CTCF, tRNA, and various insulators and binding proteins. These biological elements are often found near TAD boundary regions and are important for maintaining biological functions such as preventing the spread of heterochromatin, maintaining histone modification, and regulating transcription sites [6].

**Fig. 5:**
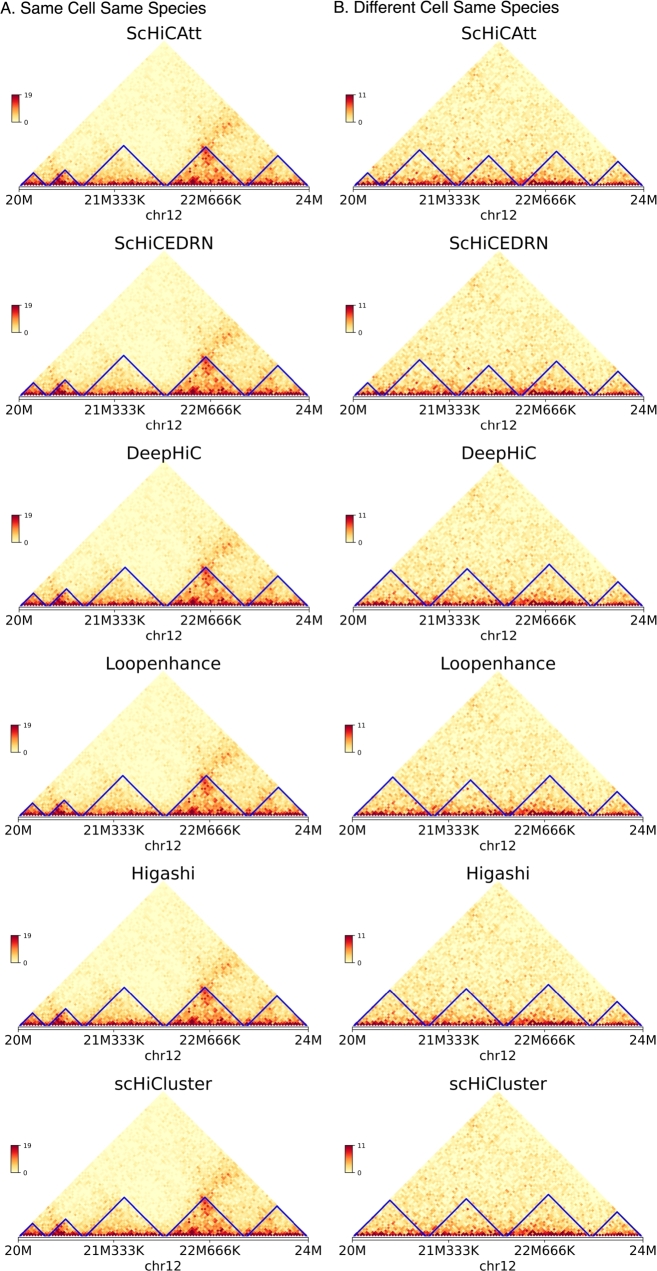
**TAD regions recovery using (A) Human cell 1 and (B) Human cell 2 for Chromosome 12 at** 40 **Kb resolution.** ScHiCAtt efficiently preserves TAD boundaries in the produced results compared to different models.

**Fig. 6:**
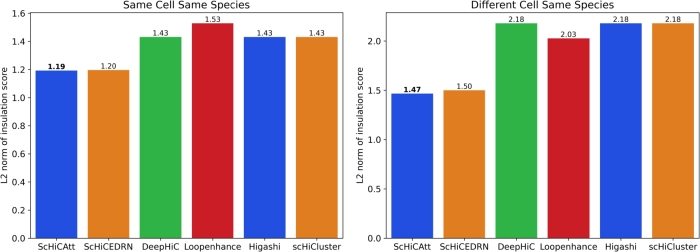
**L2 norm of TAD boundaries insulation score for (A) Human cell 1 and (B) Human cell 2.** ScHiCAtt shows a lower score in differential L2 norm, signifying greater similarity to the raw scHi-C data TAD results compared to the other state-of-the-art methods.

To validate the biological relevance of ScHiCAtt's generated results, we identified TAD regions from the result set and marked them with blue lines in Fig. 5. We compared TAD regions identified by ScHiCAtt with those from DeepHiC, ScHiCEDRN, Loopenhance, Higashi, and scHiCluster to support our model's enhanced data. We used deDoc2 [12], a graph based dynamic programming algorithm to extract TAD-like domains from the generated results, and we used lower-level TAD-like domains from the deDoc2 output to visualize TAD regions from 20 Mb to 24 Mb regions. We used the model's generated results trained with the same cell (Human Cell 1) and input these results into deDoc2 to generate and visualize TADs (Fig. 5A). ScHiCAtt preserves all the TAD information, with the predicted TADs marked by blue lines. To support ScHiCAtt's TADs, we analyzed TADs from the other five tools and visualized them. We observed that ScHiCAtt preserved TAD information comparable to the other methods, showing five TAD regions similar in number to those identified by the other tools in the specified region. To assess the robustness of ScHiCAtt, we conducted the same analysis using the model's generated results on a different cell of the same species (Human Cell 2). We visualized the TAD regions with blue lines for all four methods (Fig. 5B). We observed that ScHiCAtt preserves TAD information in the specified regions as effectively as the other methods. The similar number and lengths of TADs across all methods indicate the robustness of ScHiCAtt, regardless of the trained model used to generate the enhanced Hi-C data. To further validate our preserved TAD domains, we computed the L2 norm to quantify the similarity with the original Hi-C matrix. A lower value of the L2 norm indicates greater closeness to the original Hi-C matrix. It is challenging to find TAD boundaries from single-cell data, and to address this challenge, we calculated the insulation score as described by Zhang et al. [27] considering the TAD boundaries. Using this insulation score, we calculated the differential L2 norm of the TAD boundaries reported by ScHiCAtt, DeepHiC, Loopenhance, ScHiCEDRN, Higashi, and scHiCluster comparing them to those from the original Hi-C matrix (Fig. 6). This score reflects how closely each tool preserves the TAD domains. We observed that ScHiCAtt's L2 norm scores are 1.19 and 1.47 for the same cell and different cell scenarios, respectively. ScHiCAtt showed a lower score compared to other methods, indicating greater similarity to the original data in preserving the TAD boundary regions.

We used GenomeFlow [21] to visualize the TAD regions from 500 to 600 genomic bins to support the differential L2 norm score, as shown in Supplementary Fig. S6. We observed that ScHiCAtt's TADs are more similar to the original TADs, supporting the differential L2 norm scores of ScHiCAtt. Considering these metrics, ScHiCAtt efficiently enhances the Hi-C contact matrix while preserving biological features (e.g., TADs) across different trained models.

## 4 Discussion and conclusion

The results presented in this study demonstrate the effectiveness of the ScHiCAtt method for enhancing the resolution of single-cell Hi-C data using attention mechanisms. By experimenting with different attention configurations such as self, local, global, and dynamic attention mechanisms, ScHiCAtt achieves superior performance across several key metrics, including PSNR, SSIM, SNR, and GenomeDISCO scores, particularly at higher downsampling ratios. These results underscore the potential of attention-based models in addressing the challenges of data sparsity and resolution limitations in Hi-C data. The ScHiCAtt system demonstrates strong generalizability, as evidenced by its consistent performance across various datasets, attention mechanisms, and species, highlighting its robustness and adaptability in diverse genomic contexts. The tuning of the composite loss function significantly improved the balance between pixel-wise accuracy and structural consistency in the enhanced Hi-C contact maps, enabling ScHiCAtt to achieve superior performance across key evaluation metrics.

Furthermore, the analysis across different layers emphasizes the significance of the chosen attention mechanisms. The self-attention mechanism, while effective in capturing long-range interactions, benefits from the complementary strengths of local and global attention mechanisms. The analysis across approaches enables ScHiCAtt to balance the trade-offs between capturing fine-scale local interactions and broader, long-range genomic structures. Dynamic attention, which adjusts based on the complexity of the input, proved to be particularly effective in layers where the input signal was more variable. This suggests that a hybrid approach, where different types of attention mechanisms are applied selectively at different layers, could further enhance the performance of the model.

Additionally, the performance of ScHiCAtt across different downsampling ratios highlights its robustness and versatility. Even at lower downsampling ratios (e.g., 0.10), where data becomes increasingly sparse and challenging, ScHiCAtt maintained relatively high scores across all metrics. This resilience is particularly important for practical applications where high-resolution data is not always available, and imputation methods must be able to reconstruct accurate contact maps from limited information. The observed trend of decreasing performance with increasing downsampling ratios is consistent with expectations, as less data naturally leads to a loss of information. However, ScHiCAtt's ability to mitigate this loss better than other methods reaffirms its potential as a powerful tool for enhancing Hi-C data resolution.

Finally, as shown by the TAD analysis, TADs are useful for validating chromatin structure, but existing models often miss long-range interactions and hierarchical relationships. Our method, with integrated attention mechanisms, better captures these complex dependencies, providing more accurate and comprehensive validation by detecting TAD structures consistent with the original scHi-C data.

## Funding

This work is supported in part by the National Institutes of General Medical Sciences of the National Institutes of Health under award number R35GM150402 to O.O.

## CRediT authorship contribution statement

**Rohit Menon:** Writing – review & editing, Writing – original draft, Visualization, Validation, Software, Methodology, Investigation, Formal analysis, Data curation. **H.M.A. Mohit Chowdhury:** Writing – review & editing, Writing – original draft, Validation, Investigation, Formal analysis. **Oluwatosin Oluwadare:** Writing – review & editing, Supervision, Resources, Project administration, Funding acquisition, Formal analysis, Conceptualization.

## Declaration of Competing Interest

The authors declare that they have no known competing financial interests or personal relationships that could have appeared to influence the work reported in this paper.

## Data Availability

The ScHiCAtt project is publicly available at https://github.com/OluwadareLab/ScHiCAtt. ScHiCAtt web-server is publicly available at http://schicatt.hicrobin.online/. Drosophila Hi-C Data is publicly available at https://doi.org/10.5281/zenodo.10535486. Human Cell HiC data is available at https://salkinstitute.app.box.com/s/fp63a4j36m5k255dhje3zcj5kfuzkyj1.

## Appendix A Supplementary material

MMC This supplementary material provides additional experimental results, performance comparisons and visualizations supporting the main findings of the paper. It includes tables detailing model performance across different loss function configurations, chromosome-wise interaction statistics, and additional performance metrics across different downsampling ratios. Additionally, figures present benchmarking result for various attention mechanisms, across different cell types and species and TAD boundary visualizations. MMC

## References

[1] Ahn Namhyuk, Kang Byungkon, Sohn Kyung-Ah (2018).

[2] Arrastia Mary V. (2020).

[3] Carron Leopold (2019). Boost-HiC: computational enhancement of long-range contacts in chromosomal contact maps. Bioinformatics.

[4] Collombet Samuel (2020). Parental-to-embryo switch of chromosome organization in early embryogenesis. Nature.

[5] Dimmick Michael (2020).

[6] Dixon Jesse R. (2012). Topological domains in mammalian genomes identified by analysis of chromatin interactions. Nature.

[7] Galitsyna Aleksandra A., Gelfand Mikhail S. (2021). Single-cell Hi-C data analysis: safety in numbers. Brief Bioinform.

[8] Hicks Parker, Oluwadare Oluwatosin (2022). HiCARN: resolution enhancement of Hi-C data using cascading residual networks. Bioinformatics.

[9] Hong Hao (2020). DeepHiC: a generative adversarial network for enhancing Hi-C data resolution. PLoS Comput Biol.

[10] Huang Lun (2019). Proceedings of the IEEE/CVF international conference on computer vision.

[11] Lee Dong-Sung (2019). Simultaneous profiling of 3D genome structure and DNA methylation in single human cells. Nat Methods.

[12] Li Angsheng (2023). DeDoc2 identifies and characterizes the hierarchy and dynamics of chromatin TAD-like domains in the single cells. Adv Sci.

[13] Li Zhilan, Dai Zhiming (2020). SRHiC: a deep learning model to enhance the resolution of Hi-C data. Front Genet.

[14] Lieberman-Aiden Erez (2009). Comprehensive mapping of long-range interactions reveals folding principles of the human genome. Science.

[15] Liu Qiao, Lv Hairong, Jiang Rui (2019). hicGAN infers super resolution Hi-C data with generative adversarial networks. Bioinformatics.

[16] Liu Tong, Wang Zheng (2019). HiCNN: a very deep convolutional neural network to better enhance the resolution of Hi-C data. Bioinformatics.

[17] Liu Tong, Wang Zheng (2019). HiCNN2: enhancing the resolution of Hi-C data using an ensemble of convolutional neural networks. Genes.

[18] Oluwadare Oluwatosin, Highsmith Max, Cheng Jianlin (2019). An overview of methods for reconstructing 3-D chromosome and genome structures from Hi-C data. Biol Proc Online.

[19] Paulsen Jonas, Gramstad Odin, Collas Philippe (2015). Manifold based optimization for single-cell 3D genome reconstruction. PLoS Comput Biol.

[20] Payne Andrew C. (2021). In situ genome sequencing resolves DNA sequence and structure in intact biological samples. Science.

[21] Trieu Tuan (2019). GenomeFlow: a comprehensive graphical tool for modeling and analyzing 3D genome structure. Bioinformatics.

[22] Ulianov Sergey V. (2021). Order and stochasticity in the folding of individual Drosophila genomes. Nat Commun.

[23] Ursu Oana (2018). GenomeDISCO: a concordance score for chromosome conformation capture experiments using random walks on contact map graphs. Bioinformatics.

[24] Vaswani A. (2017). Attention is all you need. Adv Neural Inf Process Syst.

[25] Wang Yanli, Guo Zhiye, Cheng Jianlin (2023). Single-cell Hi-C data enhancement with deep residual and generative adversarial networks. Bioinformatics.

[26] Wu Qiong (2020). A novel perceptual loss function for single image super-resolution. Multimed Tools Appl.

[27] Zhang Ruochi, Zhou Tianming, Ma Jian (2022). Multiscale and integrative single-cell Hi-C analysis with Higashi. Nat Biotechnol.

[28] Zhang Shanshan (2022). DeepLoop robustly maps chromatin interactions from sparse allele-resolved or single-cell Hi-C data at kilobase resolution. Nat Genet.

[29] Zhang Yan (2018). Enhancing Hi-C data resolution with deep convolutional neural network HiCPlus. Nat Commun.

[30] Zhou Jingtian (2019). Robust single-cell Hi-C clustering by convolution- and random-walk–based imputation. Proc Natl Acad Sci.

[31] Zhu Hongyu (2021). Attention mechanisms in CNN-based single image super-resolution: a brief review and a new perspective. Electronics.

