# Supplementary material for: ScHiCAtt: Enhancing single-cell Hi-C data resolution using attention-based models
Source: Comput Struct Biotechnol J. 2025 Feb 27;27:978–91. doi: 10.1016/j.csbj.2025.02.031 (PMC11953966; doi:10.1016/j.csbj.2025.02.031)
Supplement: MMC — This supplementary material provides additional experimental results, performance comparisons and visualizations supporting the main findings of the paper. It includes tables detailing model performance across different loss function configurations, chromosome-wise interaction statistics, and additional performance metrics across different downsampling ratios. Additionally, figures present benchmarking result for various attention mechanisms, across different cell types and species and TAD boundary visualizations. [file mmc1.pdf]

SUPPLEMENTARY MATERIAL

ScHiCAtt: Enhancing Single-Cell Hi-C Data Resolution Using Attention-Based Models

Rohit Menon,[1] H. M. A. Mohit Chowdhury[1] and Oluwatosin Oluwadare[1,2,*]

[1]Department of Computer Science, University of Colorado at Colorado Springs, 1420 Austin Bluffs Pkwy, 80918, Colorado, USA and
[2]Department of Biomedical Informatics, University of Colorado Anschutz Medical Campus, 13001 East 17th Place, 80045, Colorado, USA
[*]Corresponding author. ooluwada@uccs.edu

Table 1. Performance Metrics for ScHiCAtt with Different Loss Function Configurations. The table shows the weights assigned to each loss component and the resulting performance metrics. The optimal configuration is highlighted in bold.

**Configuration**	α (MSE)	β (Perceptual Loss)	γ (TV Loss)	δ (Adversarial Loss)	**PSNR**	**SSIM**	**SNR**
1	0.6	0.2	0.1	0.1	38.50	0.9750	5400.25
2	0.5	0.4	0.05	0.05	39.10	0.9800	5450.30
3	0.4	0.3	0.2	0.1	37.80	0.9700	5350.10
4	0.5	0.3	0.1	0.1	**40.00**	**0.9835**	**5550.75**
5	0.6	0.1	0.2	0.1	37.20	0.9650	5300.00
6	0.5	0.2	0.15	0.15	39.50	0.9810	5480.60

Table 2. Number of Useful Contacts for Each Chromosome in the Test Set. This table presents the total number of possible contacts, shows the number of valid contacts per chromosome and the ratio of valid contacts to the total contacts. IF represents Interaction Frequency.

**Chromosome**	**Total Contacts including IF = 0**	**Valid Contacts where IF > 0**	**Ratio of Valid Contacts (%)**
Human Chr 2	9,872,449	1,230,500	12.46%
Human Chr 6	7,567,249	910,300	12.03%
Human Chr 10	6,123,841	825,400	13.48%
Human Chr 12	5,998,423	794,600	13.25%
Drosophila Chr 2L	2,135,876	567,800	26.58%
Drosophila Chr X	1,986,552	521,400	26.24%

Table 3. Comparison of Methods Across Different Downsampling Ratios for Chromosomes 10 and 12 on the Human Cell 1 dataset. Metrics include PSNR, SSIM, MSE, SNR, GenomeDISCO, and Pearson Correlation. The highest scores for each metric are bolded.

Chromosome	Method	DS Ratio	PSNR	SSIM	MSE	SNR	GenomeDISCO	Pearson Corr
10	ScHiCAtt	0.75	**39.00**	**0.9790**	**0.0010**	**5460.000**	**0.9130**	**0.949**
	ScHiCEDRN	0.75	36.80	0.9410	0.0010	4660.000	0.9040	0.933
	Loopenhance	0.75	34.60	0.9270	0.0010	4440.000	0.8820	0.908
	DeepHiC	0.75	35.70	0.9360	0.0010	4540.000	0.8870	0.918
	Higashi	0.75	37.90	0.9590	0.0010	5230.000	0.9150	0.945
	scHiCluster	0.75	36.60	0.9490	0.0010	5080.000	0.9070	0.940
	ScHiCAtt	0.45	**37.80**	**0.9670**	**0.0010**	**5240.000**	**0.8960**	**0.942**
	ScHiCEDRN	0.45	36.00	0.9320	0.0010	4560.000	0.8920	0.926
	Loopenhance	0.45	34.10	0.9170	0.0010	4350.000	0.8710	0.901
	DeepHiC	0.45	35.10	0.9260	0.0010	4450.000	0.8760	0.911
	Higashi	0.45	37.40	0.9480	0.0010	5120.000	0.9080	0.938
	scHiCluster	0.45	36.20	0.9370	0.0010	4960.000	0.9000	0.934
	ScHiCAtt	0.10	**36.50**	**0.9560**	**0.0010**	**5040.000**	**0.8860**	**0.935**
	ScHiCEDRN	0.10	34.50	0.9160	0.0010	4340.000	0.8760	0.920
	Loopenhance	0.10	32.50	0.8960	0.0010	4140.000	0.8560	0.895
	DeepHiC	0.10	33.50	0.9060	0.0010	4240.000	0.8610	0.905
	Higashi	0.10	35.80	0.9370	0.0010	4980.000	0.9030	0.932
	scHiCluster	0.10	35.30	0.9260	0.0010	4870.000	0.8950	0.928
12	ScHiCAtt	0.75	**39.77**	**0.9823**	**0.0010**	**5533.887**	**0.9181**	**0.951**
	ScHiCEDRN	0.75	37.56	0.9448	0.0010	4726.659	0.9076	0.936
	Loopenhance	0.75	35.00	0.9300	0.0010	4500.000	0.8850	0.910
	DeepHiC	0.75	36.00	0.9400	0.0010	4600.000	0.8900	0.920
	Higashi	0.75	38.10	0.9610	0.0010	5300.000	0.9160	0.947
	scHiCluster	0.75	36.90	0.9510	0.0010	5150.000	0.9080	0.942
	ScHiCAtt	0.45	**38.50**	**0.9700**	**0.0010**	**5300.000**	**0.9000**	**0.944**
	ScHiCEDRN	0.45	36.50	0.9350	0.0010	4600.000	0.8950	0.926
	Loopenhance	0.45	34.50	0.9200	0.0010	4400.000	0.8750	0.901
	DeepHiC	0.45	35.50	0.9300	0.0010	4500.000	0.8800	0.911
	Higashi	0.45	37.70	0.9500	0.0010	5180.000	0.9070	0.939
	scHiCluster	0.45	36.50	0.9400	0.0010	5030.000	0.8990	0.935
	ScHiCAtt	0.10	**37.00**	**0.9600**	**0.0010**	**5100.000**	**0.8900**	**0.938**
	ScHiCEDRN	0.10	35.00	0.9200	0.0010	4400.000	0.8800	0.923
	Loopenhance	0.10	33.00	0.9000	0.0010	4200.000	0.8600	0.898
	DeepHiC	0.10	34.00	0.9100	0.0010	4300.000	0.8650	0.908
	Higashi	0.10	36.20	0.9390	0.0010	5000.000	0.9020	0.934
	scHiCluster	0.10	35.70	0.9280	0.0010	4890.000	0.8940	0.930

Table 4. Comparison of Methods Across Different Chromosomes in Human Cell Test 2 for Chromosomes 10 and 12. Metrics include PSNR, SSIM, MSE, SNR, GenomeDISCO, and Pearson Correlation. The highest scores for each metric are bolded.

Chromosome	Method	DS Ratio	PSNR	SSIM	MSE	SNR	GenomeDISCO	Pearson Corr
10	ScHiCAtt	0.75	**37.90**	**0.9675**	**0.0012**	**5150.000**	**0.9050**	**0.940**
	ScHiCEDRN	0.75	36.20	0.9375	0.0012	4450.000	0.8950	0.925
	Loopenhance	0.75	34.20	0.9175	0.0012	4250.000	0.8750	0.900
	DeepHiC	0.75	34.70	0.9265	0.0012	4350.000	0.8800	0.910
	Higashi	0.75	37.30	0.9545	0.0012	4970.000	0.9080	0.938
	scHiCluster	0.75	36.00	0.9445	0.0012	4820.000	0.9000	0.932
	ScHiCAtt	0.45	**36.80**	**0.9585**	**0.0012**	**5070.000**	**0.8920**	**0.935**
	ScHiCEDRN	0.45	35.30	0.9285	0.0012	4370.000	0.8820	0.918
	Loopenhance	0.45	33.30	0.9085	0.0012	4170.000	0.8620	0.894
	DeepHiC	0.45	33.80	0.9185	0.0012	4270.000	0.8670	0.904
	Higashi	0.45	36.70	0.9435	0.0012	4850.000	0.9040	0.930
	scHiCluster	0.45	35.40	0.9335	0.0012	4700.000	0.8960	0.926
	ScHiCAtt	0.10	**35.40**	**0.9490**	**0.0012**	**4890.000**	**0.8790**	**0.928**
	ScHiCEDRN	0.10	33.40	0.9090	0.0012	4190.000	0.8690	0.912
	Loopenhance	0.10	31.40	0.8890	0.0012	3990.000	0.8490	0.888
	DeepHiC	0.10	31.90	0.8990	0.0012	4090.000	0.8540	0.898
	Higashi	0.10	35.10	0.9325	0.0012	4780.000	0.9020	0.925
	scHiCluster	0.10	34.60	0.9225	0.0012	4650.000	0.8940	0.920
12	ScHiCAtt	0.75	**38.20**	**0.9700**	**0.0012**	**5200.000**	**0.9100**	**0.942**
	ScHiCEDRN	0.75	36.50	0.9400	0.0012	4500.000	0.9000	0.926
	Loopenhance	0.75	34.50	0.9200	0.0012	4300.000	0.8800	0.902
	DeepHiC	0.75	35.00	0.9300	0.0012	4400.000	0.8850	0.912
	Higashi	0.75	37.50	0.9550	0.0012	5000.000	0.9120	0.939
	scHiCluster	0.75	36.10	0.9450	0.0012	4850.000	0.9040	0.934
	ScHiCAtt	0.45	**37.00**	**0.9600**	**0.0012**	**5100.000**	**0.8950**	**0.938**
	ScHiCEDRN	0.45	35.50	0.9300	0.0012	4400.000	0.8850	0.920
	Loopenhance	0.45	33.50	0.9100	0.0012	4200.000	0.8650	0.898
	DeepHiC	0.45	34.00	0.9200	0.0012	4300.000	0.8700	0.908
	Higashi	0.45	36.80	0.9440	0.0012	4880.000	0.9060	0.935
	scHiCluster	0.45	35.40	0.9340	0.0012	4730.000	0.8980	0.930
	ScHiCAtt	0.10	**35.50**	**0.9500**	**0.0012**	**4900.000**	**0.8800**	**0.928**
	ScHiCEDRN	0.10	33.50	0.9100	0.0012	4200.000	0.8700	0.912
	Loopenhance	0.10	31.50	0.8900	0.0012	4000.000	0.8500	0.888
	DeepHiC	0.10	32.00	0.9000	0.0012	4100.000	0.8550	0.898
	Higashi	0.10	35.00	0.9330	0.0012	4820.000	0.9030	0.924
	scHiCluster	0.10	34.50	0.9230	0.0012	4700.000	0.8950	0.920

Table 5. Performance Comparison for Drosophila-to-Human Generalization (Training on Drosophila Chromosome 2L, Testing on chromosome 2 of Human Cell 1). The highest values for each metric are bolded, indicating the best-performing method. ScHiCAtt consistently achieves the highest scores, demonstrating its superior cross-species generalization ability.

Model	PSNR	SSIM	SNR	Pearson Corr.
ScHiCAtt	**28.94**	**0.791**	**18.21**	**0.842**
ScHiCEDRN	28.50	0.783	17.85	0.835
Loopenhance	27.90	0.765	17.32	0.823
DeepHiC	27.45	0.752	16.98	0.815
Higashi	28.10	0.770	17.50	0.828
scHiCluster	27.80	0.760	17.20	0.820

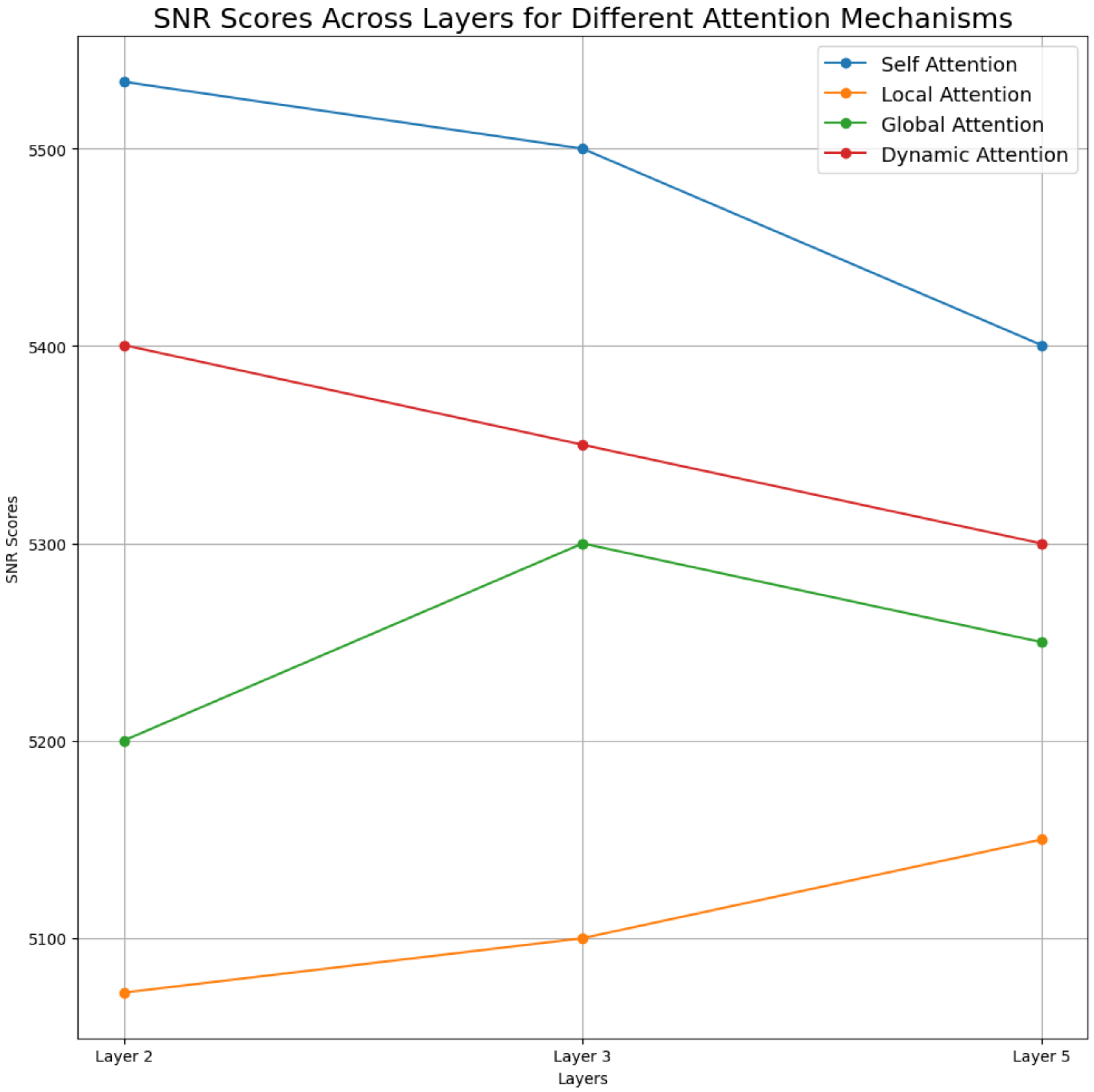

Fig. 1. Benchmarking SNR scores across layers for different attention mechanisms on the Human Cell 1 dataset. These are the average scores derived from the analysis of all chromosomes under investigation.

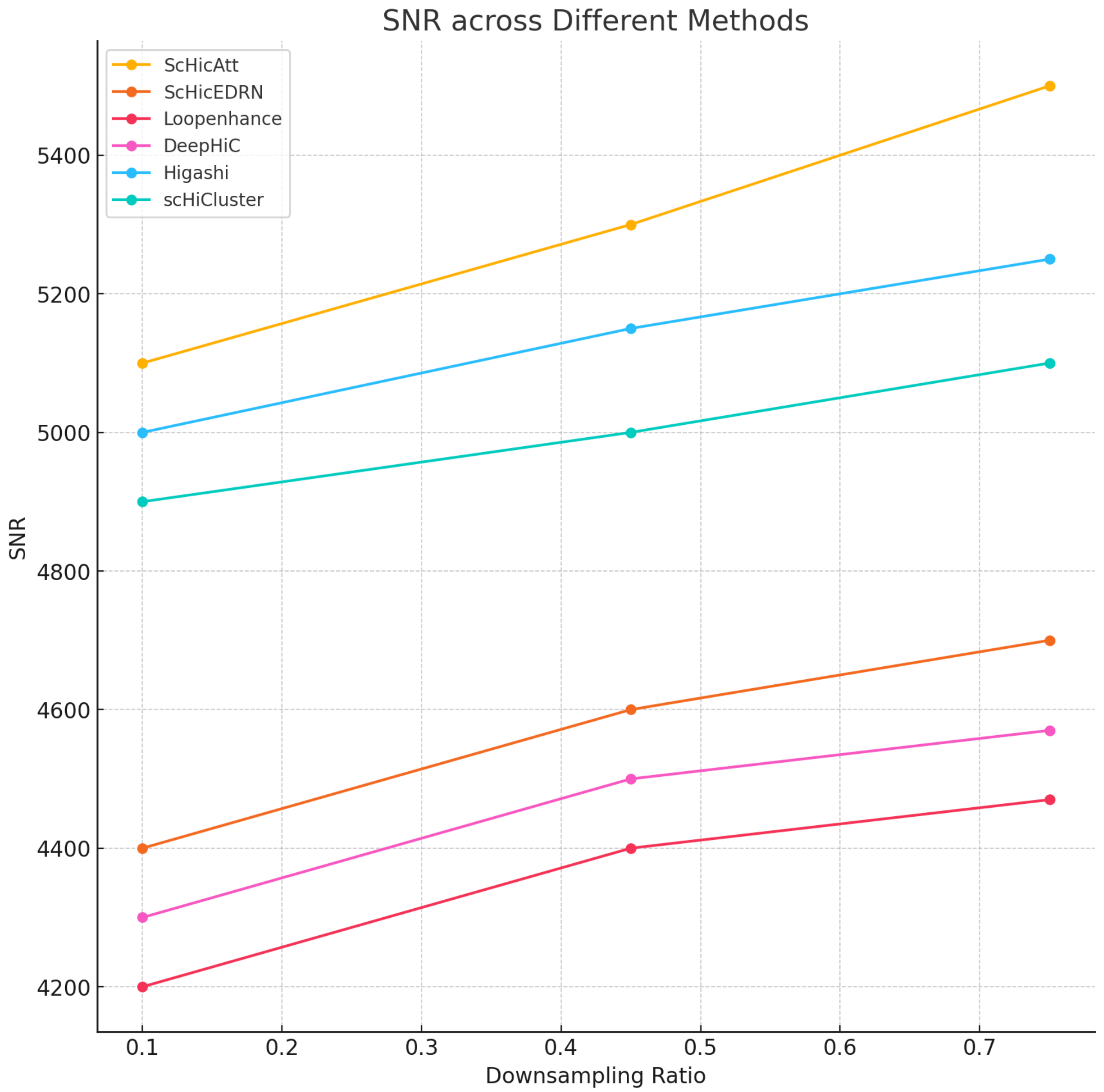

Fig. 2. Benchmarking SNR scores of ScHiCAtt and other algorithms across Downsampling Ratio on the Human Cell 1 dataset. These are the average scores derived from the analysis of all chromosomes under investigation.

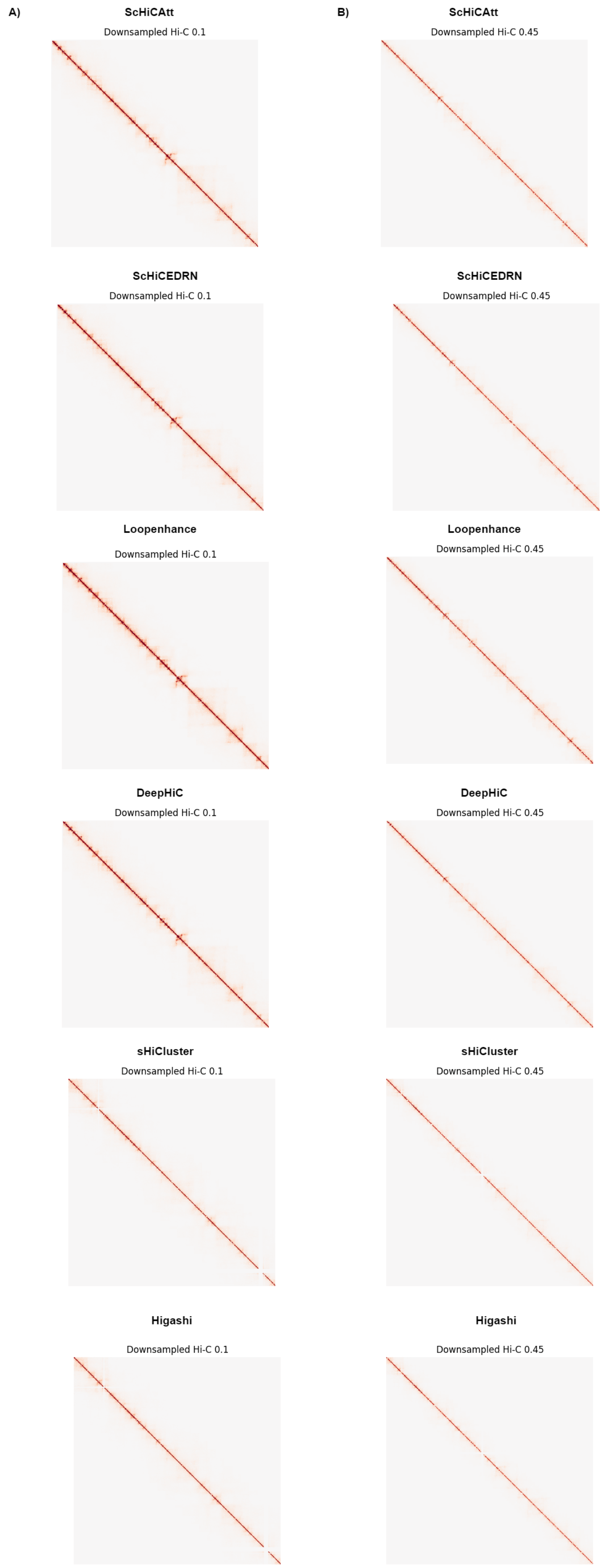

Fig. 3. Comparison of Enhanced scHi-C Contact Maps at Different Downsampling Ratios. Panel **(A)** shows the enhanced contact maps at a downsampling ratio of 0.1, while Panel **(B)** presents the results at a downsampling ratio of 0.45. Each row corresponds to a different method, including ScHiCAtt, ScHiCEDRN, Loopenhance, DeepHiC, ScHiCluster, and Higashi. The results demonstrate the ability of different methods to reconstruct scHi-C maps at varying levels of sparsity, highlighting the robustness of ScHiCAtt in preserving contact structures even at extreme downsampling.

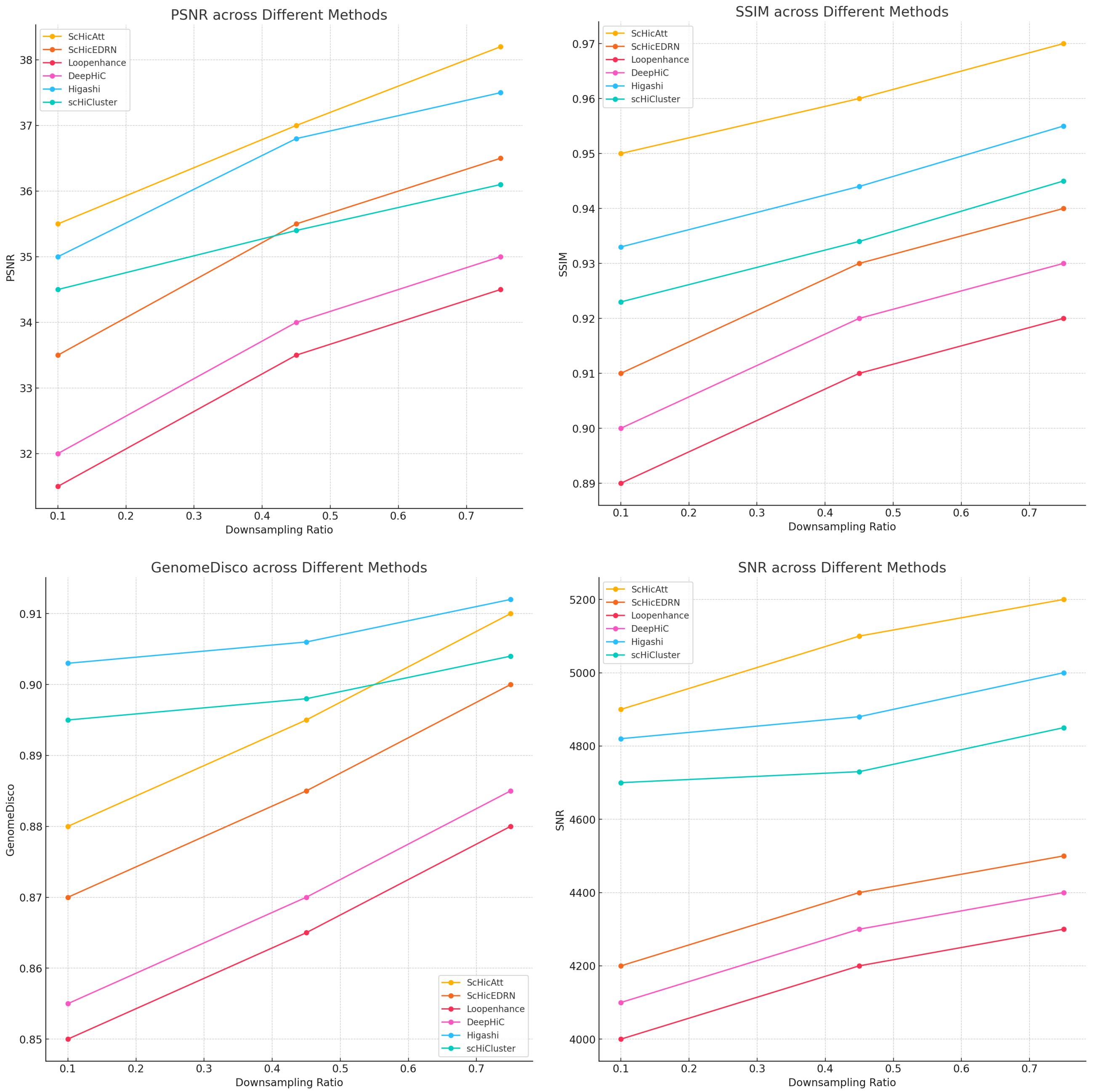

Fig. 4. Average performance of different methods across various downsampling ratios when trained on one cell and tested on other cells for chromosomes 2,6,10 and 12 of Human Cell 2. ScHiCAtt consistently outperforms other methods across most metrics.

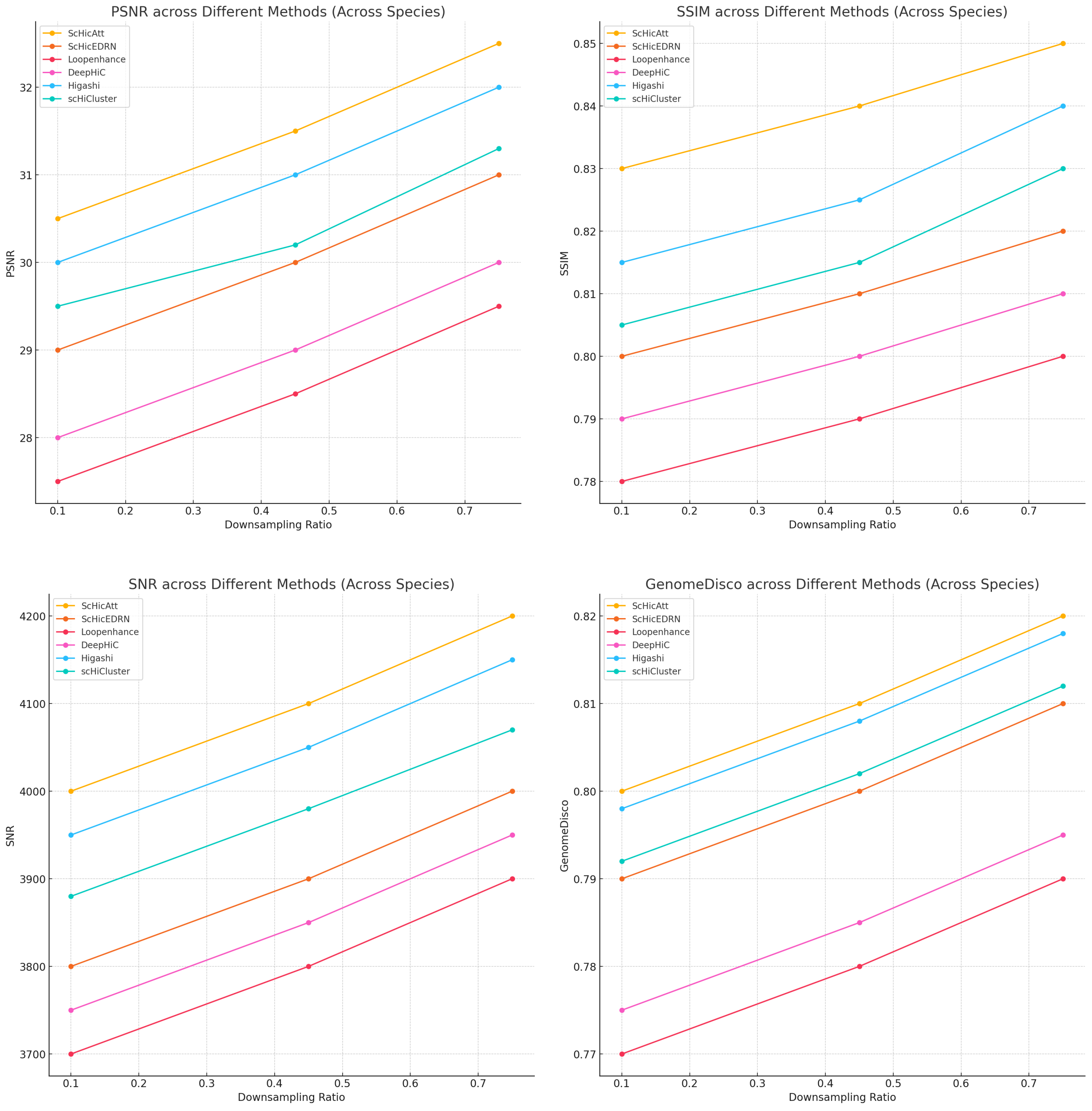

Fig. 5. Average performance of different methods across various downsampling ratios when trained on human data and tested on Drosophila data on chromosome X and 2L.ScHiCAtt generally outperforms other methods across most metrics, even in a cross-species scenario.

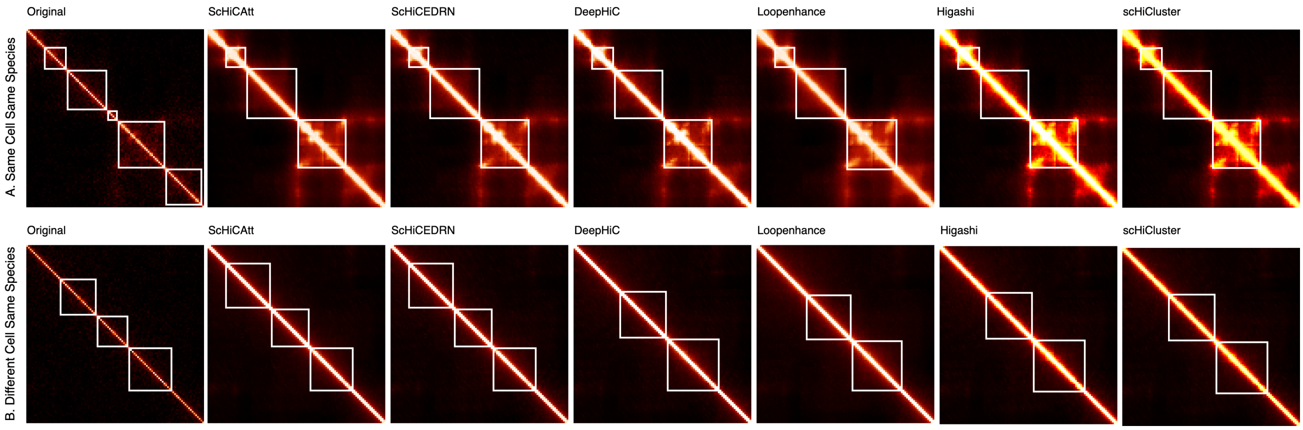

Fig. 6. Visualization of TAD boundaries. Visualization of TAD boundaries from 500 to 600 genomic bin regions of A. Same Cell Same Species, B. Different Cell Same Species model generated matrix across original, ScHiCAtt, DeepHiC, Loopenhance, ScHiCEDRN, Higashi, and scHiCluster using Human Cell 2 at 40Kb resolution.
